# Pigment Dispersing Factors and Their Cognate Receptors in a Crustacean Model, With New Insights Into Distinct Neurons and Their Functions

**DOI:** 10.3389/fnins.2020.595648

**Published:** 2020-10-29

**Authors:** Jodi L. Alexander, Andrew Oliphant, David C. Wilcockson, Timothy Brendler-Spaeth, Heinrich Dircksen, Simon G. Webster

**Affiliations:** ^1^School of Natural Sciences, Brambell Laboratories, Bangor University, Bangor, United Kingdom; ^2^Institute of Biological Environmental and Rural Sciences, Edward Llwyd Building, Aberystwyth University, Aberystwyth, United Kingdom; ^3^Department of Biochemistry, University of Otago, Dunedin, New Zealand; ^4^Department of Zoology, Stockholm University, Stockholm, Sweden

**Keywords:** pigment dispersing hormone, G protein-coupled receptor deorphaning, neuroanatomy, gene expression, functions

## Abstract

Pigment dispersing factors (PDFs, or PDHs in crustaceans) form a structurally related group of neuropeptides found throughout the Ecdysozoa and were first discovered as pigmentary effector hormones in crustaceans. In insects PDFs fulfill crucial neuromodulatory roles, most notably as output regulators of the circadian system, underscoring their central position in physiological and behavioral organization of arthropods. Intriguingly, decapod crustaceans express multiple isoforms of PDH originating from separate genes, yet their differential functions are still to be determined. Here, we functionally define two PDH receptors in the crab *Carcinus maenas* and show them to be selectively activated by four PDH isoforms: PDHR 43673 was activated by PDH-1 and PDH-2 at low nanomolar doses whilst PDHR 41189 was activated by PDH-3 and an extended 20 residue e-PDH. Detailed examination of the anatomical distribution of all four peptides and their cognate receptors indicate that they likely perform different functions as secreted hormones and/or neuromodulators, with PDH-1 and its receptor 43,673 implicated in an authentic hormonal axis. PDH-2, PDH-3, and e-PDH were limited to non-neurohemal interneuronal sites in the CNS; PDHR 41189 was largely restricted to the nervous system suggesting a neuromodulatory function. Notably PDH-3 and e-PDH were without chromatophore dispersing activity. This is the first report which functionally defines a PDHR in an endocrine system in a crustacean and to indicate this and other putative roles of this physiologically pivotal peptide group in these organisms. Thus, our findings present opportunities to further examine the endocrine and circadian machinery in this important arthropod phylum.

## Introduction

Pigment dispersing factors (PDFs) form a group of conserved, structurally related neuropeptides in ecdysozoans. They were first described as color-change hormones in crustaceans in view of their (neurohormonal) functions in chromatic adaptation by dispersing epidermal chromatophores (Kleinholz, 1975) and distal retinal pigments (Fernlund, 1976) [reviews; (Rao, 2001; Rao and Riehm, 2001)], hence the appellation pigment dispersing hormones (PDHs). In crustaceans, PDH mediated circadian rhythms of pigment dispersion in the compound eye and chromatophores have long been established [review; (Strauss and Dircksen, 2010)]. Electroretinograms (ERG) of crayfish, which show clear circadian rhythms (Aréchiga et al., 1993; Aréchiga and Rodríguez-Soza, 1998) can be phase-set by PDH injection (Verde et al., 2007). Interestingly, some PDH immunoreactive interneurons in the water flea *Daphnia magna*, undergo circadian variation in immunolabelling intensity and appear to be homologs of well-established neurons exhibiting circadian rhythmicity in *Drosophila melanogaster* (Strauss et al., 2011). Thus, it seems likely that this peptide is a key component of the circadian clockwork in crustaceans.

A PDH-like peptide was identified from an insect, *Romalea microptera* on the basis of a chromatophore dispersion bioassay in a crustacean (Rao et al., 1987), and a very large number of PDH/PDF peptides and pre-processed precursors have now been conceptually identified from transcriptomes and genomes of arthropods [see (Mayer et al., 2015) for a comprehensive list]. It is well established that these peptides fulfill a wide variety of functions as neuromodulators/transmitters in insects, but notably, are of fundamental importance as circadian clock output factors, controlling daily rhythms in locomotor activity [e.g., (Renn et al., 1999; Helfrich-Förster et al., 2000; Helfrich-Förster, 2009)]. Further diverse functions in controlling geotactic behavior, ureter contractions, arousal, and reproduction have also been identified in several insect species [review: (Meelkop et al., 2011)].

PDF-related peptides that bear significant N-terminus identity with arthropod PDFs have been found in nematodes; in *Caenorhabditis elegans* two *pdf* genes encode three different peptides, two of which (PDF1a,b) have sufficient sequence identity to display functional cross reactivity in a crustacean chromatophore bioassay (Meelkop et al., 2012). PDF-1 mutants mimic the behavioral phenotype of *Drosophila pdf-*null mutants, i.e., disruption of free-running locomotor rhythms (Janssen et al., 2009). Since *pdf* homologs have been mined from the transcriptomes of several species of onychophorans and tardigrades (Christie et al., 2011; Mayer et al., 2015), and PDH-immunopositive neurons have been mapped in pulmonate gastropods (Elekes and Nässel, 1999), an attractive hypothesis is that PDF-like molecules have a wide distribution in invertebrates. Indeed, there is a limited identity of these molecules to “cerebrin-type” peptides in *Aplysia californica* (Li et al., 2011), several lophotrochozoans (Veenstra, 2010, 2011), deuterostomes such as starfish, sea urchins, (Semmens et al., 2016) and a hemichordate (Mirabeau and Joly, 2013). These, and many other studies, have reinforced the emerging scenario by which orthologous neuropeptide families can be traced back to the common ancestor of the Bilateria (Jekely, 2013; Elphick et al., 2018).

The cognate receptor for PDF has been deorphaned, using a reverse pharmacology approach, and functionally characterized in *Drosophila* (Hyun et al., 2005; Lear et al., 2005; Mertens et al., 2005). This approach also identified three different splice PDFR isoforms in *C. elegans* that can be activated by all three PDFs in the heterologous assay used (Janssen et al., 2008). These studies showed that PDFRs are class B (secretin) G-protein coupled receptors (GPCRs) that signal via cAMP and are related to the mammalian vasoactive intestinal peptide receptor (VPAC2) and calcitonin receptors. This is noteworthy, since these are expressed by the mammalian circadian clock. In *Drosophila pdfr* mutant’s phenocopy *pdf* null mutants in terms of aberrant behavioral rhythmicity and have a severe negative geotaxis phenotype (Mertens et al., 2005).

PDH receptors have not yet been functionally characterized in any crustacean despite tentative annotation by sequence homology to the *Drosophila* PDFR and a number of other insect putative PDFRs. Annotations include those for crabs: *Scylla paramamosain* (Bao et al., 2018), *Gecarcinus lateralis* (Tran et al., 2019) lobster: *Homarus americanus* (Christie et al., 2015), shrimp: *Marsupenaeus japonicus* (Asazuma-Mabashi et al., unpublished) and water flea: *Daphnia pulex* (Colbourne et al., 2011). Furthermore, in crustaceans, multiple isoforms (2–3) of PDH in a single species have been documented (Klein et al., 1994; Rao, 2001; Hsu et al., 2008; Mayer et al., 2015), and it has long been known that crustaceans, as alluded to earlier, use PDH to regulate several physiological functions via neurohormonal and/or neurotransmitter modalities. This situation is in contrast to insects where a single PDH isoform has been reported.

Our initial screen of neurotranscriptomes from the green shore crab, *Carcinus maenas* (Oliphant et al., 2018) revealed three candidate PDHRs, and four PDH peptides. This led us to develop a working hypothesis involving the diversification of systemic neurohormonal PDH signaling and that involving neuromodulatory functions via a neurotransmitter-type role. Thus, we set out to answer the fundamental question–which ligand receptor pairings are associated with a distinct function? For the first time in crustacean endocrinology, we have used a combined approach to identify ligand/receptor pairing, receptor and ligand expression, and bioassay to identify two functional PDHRs that are selectively activated by PDHs which have either neurohormonal and/or neurotransmitter function associated with chromatic adaptation and likely circadian rhythmicity.

## Materials and Methods

### Animals and Tissue Collection

Specimens of mature green shore crab, *C*. *maenas* were collected using baited traps from the Menai Strait, United Kingdom. Crabs were maintained in a recirculating seawater system at ambient temperature and photoperiod and fed with fish. Nervous system tissues from intermolt crabs were dissected, following anesthesia on ice, and processed either for immunohistochemistry (IHC), with Stefanini’s fixative (Stefanini et al., 1967), (overnight, 4^o^C), or for *in situ* hybridization (ISH), 4% paraformaldehyde (PFA) in phosphate buffered saline (PBS), overnight at 4^o^C. For RNA extractions, tissues were dissected, immediately frozen in liquid nitrogen, and stored at −80^o^C.

### Transcriptome Data Mining

Transcriptome sequencing of neural tissue and data mining was performed as described elsewhere (Oliphant et al., 2018). Contigs mined as putative neuropeptide receptor sequences were translated using ExPASy translate (Artimo et al., 2012), submitted to tBLASTn searches against the NCBI database, and transmembrane domains predicted using TMHMM server v2.0 (Sonnhammer et al., 1988). Phylograms of putative PDFRs were assembled using Geneious V8 Tree builder, (Jukes-Cantor model, default setting for neighbor joining).

### Quantitative and RT-PCR, Cloning, and Sequencing

Tissue specific transcript expression of the PDH receptors (PDHR) was performed using Taqman MGB hydrolysis probes as previously described (Hoelters et al., 2016). The production of standard RNAs and reverse transcription of standards and unknowns were performed as described previously (Alexander J. L. et al., 2018). Duplex qPCR reactions (10 μl) which simultaneously amplified target and reference genes were performed in triplicate using Sensifast Probe II reagents (Bioline, United Kingdom) and run on an Applied Biosystems QuantStudio 12-Flex machine. Expression values were calculated according to the 2^–Δ^ ^Δ^ ^C^_*T*_ method (Livak and Schmittgen, 2001), normalized to the geometric mean of the stably expressed *elongation factor-1* and *ubiquitin*-*conjugating enzyme E2* L3 (Oliphant et al., 2018). Cerebral ganglia samples were used as the calibrator. Primer sequences are shown in Supplementary Table 1.

Standard RT-PCR was used to determine the expression of PDH transcripts in the CNS (eyestalk, cerebral ganglion, connective ganglion, ventral ganglion) and chela muscle control. Total RNA was extracted with TRIzol (Ambion, Carlsbad CA, United Kingdom), gDNA removed with Turbo DNA-free (Ambion), and 5 μg quantities of RNA purified for mRNA selection using Dynabeads (Dynal AS, Oslo, Norway). The resulting mRNA was reverse transcribed using a Tetro cDNA synthesis kit (Bioline, United Kingdom), with a 1:1 mixture of random/oligo dT primers. cDNA was diluted 10-fold and standard PCR performed using Platinum II hot start enzyme mix (Invitrogen, Thermo Fisher Scientific Vilnius, Lithuania). Conditions: denature/activation 94^o^C 30 s, 32 cycles 63^o^C 30 s, 72^o^C 30 s, extension 72^o^C 5 min. Primer sequences are shown in Supplementary Table 1.

To confirm the correct sequence identity of the 4 PDH’s identified in the neurotranscriptomes (PDH-1-3, e-PDH) and obtain the UTR sequences, 3′ and 5′ RLM RACE (GeneRacer, Invitrogen) was performed using eyestalk (PDH-1-3) and cerebral ganglion (e-PDH) cDNA followed by cloning and sequencing as previously detailed (Wilcockson et al., 2011).

### PDH Receptor Assays

#### Isolation and Cloning of PDH Receptors

Neural tissue transcriptome mining revealed three candidates encoding putative PDHRs (annotated: 35701, 41189, and 43673), based on sequence similarity with several insect PDFRs. PCR of these transcripts was performed as described previously (Alexander J. et al., 2018; Alexander J. L. et al., 2018). Primer sequences for directional cloning and expression are shown in Supplementary Table 1. Correctly sized PCR products were directionally cloned into pcDNA 3.1 D/V5-His-TOPO plasmids (Invitrogen) and the recombinant vector transformed into TOP 10 competent cells (Invitrogen). Positive clones were cultured overnight in selective LB medium with 100 μg ml^–1^ ampicillin, plasmids extracted (FastPlasmid Mini Kit, 5Prime, Hamburg, Germany), resequenced to confirm insert orientation and verification (MWG Eurofins, Ebersberg, Germany), and analyzed using Geneious V 9.1.8.

#### Receptor Assays

A heterologous cell–based assay using Chinese hamster ovary cells (CHO-K1) expressing apoaequorin (Perkin Elmer, Boston, MA, United Kingdom) and either the Gα16 or G*_*q*_* subunit (control cells) was used to report intracellular Ca^2+^ fluxes after exposure to ligand, as previously detailed (Alexander J. et al., 2018; Alexander J. L. et al., 2018).

Peptides used in the luminescence assay were synthetic *C*. *maenas* PDH-1-3 and e-PDH, DH-31 (Genecust Dudelange, Luxembourg). Peptides were reconstituted in 30% acetonitrile, aliquoted and dried by vacuum centrifugation and subsequently re-dissolved in BSA medium (DMEM/F, 50 mM HEPES, 0.1% BSA) immediately prior to assay, and dispensed into quadruplicate wells of white 96 well plates (OptiPlate, Perkin Elmer). Cell suspensions (5 × 10^6^ cells/ml) were stirred gently and 50 μl amounts were injected into each well using a Mithras LB940 microplate reader (Berthold Technologies, Bad-Wildbad, Germany). Ca^2+^ evoked luminescence was recorded for 40 s, followed by cell lysis (injection of 0.3% Triton-X 100 in BSA medium), and light emission recorded for a further 10 s. BSA medium was used for blank measurements (6 replicates per plate), and mock transfections with empty vectors for negative controls. Data reduction and analysis was done using MikroWin v5.18 (Mikrotek Laborsysteme, GmBH, Overath, Germany) and SigmaPlot v.13 (Systat Software Inc., San José CA, United States). Receptor responses were normalized against total Ca^2+^ luminescence.

### PDH Bioassays

The pigmentary effector activity of all PDHs was measured *in vivo* using small (15–20 mm carapace width) *C. maenas*, which were light adapted against a white background (300 lux for 1 h). Pigment dispersion of the red and black chromatophores in the dactyl of the last walking leg was observed microscopically and indices of dispersion scored (Hogben and Slome, 1931). Peptides were reconstituted in physiological saline (Saver et al., 1999) immediately prior to assay, to minimize Met oxidation (storage of reconstituted peptides in frozen saline for extended periods completely abolished biological activity) and injected (10 μl) using hand-drawn, calibrated microcapillaries at 50–0.05 pmol per crab, with saline controls. Chromatophore dispersion indices were scored at 20 min intervals for 1 h.

### Immunohistochemistry and *in situ* Hybridization

Fixed nervous systems were processed for whole mount IHC as previously described (Webster et al., 2013). In some, whole eyestalks, including cuticle were taken from postmolt crabs, and routinely fixed (Bouin’s) and processed for paraffin wax embedding; thick serial sections (25–30 μm) were then prepared and processed for IHC. Primary antiserum concentrations [anti-PDH code R171, raised to a thyroglobulin conjugate of PDH-1 as described (Dircksen et al., 1987) and characterized (Wilcockson et al., 2011)] was 1:2000. Secondary antiserum dilution (Alexa Fluor 488 goat anti-rabbit, Invitrogen, Thermo Fisher Scientific) was 1:750. Preparations were mounted on cavity slides using Vectashield (VectorLabs, United Kingdom), cover slipped, sealed with nail varnish, and images collected and Z-stacked at 5 μm intervals on a Zeiss 710 confocal microscope equipped with Zen Black edition software (Carl Zeiss, Jena, Germany).

Whole mount *in situ* hybridizations were performed using digoxygenin (DIG)-labeled riboprobes produced for each PDH, as previously described (Webster et al., 2013). DIG-labeled antisense run-off probes were synthesized using primers detailed in Supplementary Table 1 (NB: specificity tests using sense constructs did not give any hybridization signals). Preparations were mounted on cavity slides in 50% glycerol/PBS and sealed. Images were cropped, resized and adjusted for brightness and contrast using Adobe Photoshop CC2017 and CorelDraw 2014.

### HPLC, Immunoassay and Mass Spectrometry

To identify the neurohormonal PDH inventory, 50 sinus glands (SG) were extracted in 2 M acetic acid and separated by HPLC. Conditions: 4.6 mm × 300 mm Jupiter C18 300 Å column (Phenomenex, Macclesfield, United Kingdom) 40–80% solvent B over 30 min, 1 ml/min, detection at 210 nm, solvent A, 0.11% trifluoroacetic acid (TFA); solvent B 60% acetonitrile, 0.1% TFA. Fractions (1 ml) were dried by vacuum centrifugation and reconstituted in 0.1 M bicarbonate buffer, pH 9.3 immediately prior to immunoassay. Standard PDHs (800 pmol) were subsequently chromatographed to establish SG fractions for further MS analysis. PDH immunoreactive fractions were identified by enzyme immunoassay (EIA). Reconstituted fractions (100 μl) were coated onto high protein binding microplates (Costar 3590, Corning, VWR International, United Kingdom), overnight at 4^o^C, followed by washing (3 times with 0.1 M bicarbonate buffer), blocking (0.1% BSA, 1 h RT) and incubation in 100 μl/well 1:2000 anti-PDH in containing 0.1% Tween 20 (PBST), overnight 4^o^C. After washing (5 times with PBST), plates were incubated in 1:5000 goat anti-rabbit peroxidase labeled IgG (Vector laboratories, United Kingdom), for 6 h, RT. After washing (5 times with PBST), peroxidase activity was visualized (0.04% ABTS, 0.01% H_2_O_2_ (30%), 0.1 M phosphate/citrate buffer, pH 4.0). PDH-1 2500-2.5 fmol per well was used to generate standard curves for quantification. Absorbance at 405 nm was measured on a microplate reader (Mithras LB 940) with data analysis (MikroWin v5.18).

Fractions (25 SG equivalents) corresponding to each PDH were mass analyzed by LC MS/MS on an Orbitrap Fusion Tribrid mass spectrometer (Thermo Fisher Scientific, United Kingdom) delivered by an UltiMate 3000 UHPLC liquid chromatography platform (Dionex, Thermo Fisher Scientific, United Kingdom). Samples and standards were reconstituted in 20 μl 0.1% formic acid and 10 μl separated on a Zorbax Eclipse Plus (Agilent, Santa Clara, CA, United Kingdom) reverse phase C18 column (50 mm × 2.1 mm; particle size 1.8 μm), 30°C. Gradient elution conditions were: ultra-pure water (18.2Ω) containing 0.1% formic acid, (Solvent A) and 95:5 acetonitrile: water with 0.1% formic acid (solvent B) from 3–40% B over 9 min followed by an increase to 100% B over 2 min before holding for 1 min at 100% B. Flow rate 0.1 ml/min.

Ions were generated in an H-ESI source with a source voltage of 3500 in positive mode, sheath gas: 25, aux gas: 5, a vaporizer temperature of 75°C and an ion transfer temperature of 275°C. Standard peptide analysis parameters for a data dependent MS/MS experiment were used. Parent ions were detected in profile mode in the range 375–1500 in the Orbitrap at a resolution of 1,20,000 and a maximum injection time of 50 ms in positive mode. MS/MS data were recorded in data dependent mode including charge states of 2–7. Dynamic exclusion of masses for 20 s after initial selection for MS/MS was conducted. Ions were generated via fragmentation by collision-induced dissociation with a collision energy of 35% and detected in the Ion Trap in centroid mode. Peak lists were exported as Mascot Generic Files and used to search against the *C. maenas* CNS transcriptome (Oliphant et al., 2018) using the MASCOT program (Matrix Science Ltd., United Kingdom, version 2.1) with a default peptide score of 50 used to determine significance. Search parameters allowed a maximum of one missed cleavage. Variable modifications tested for matches with 0, 1, 2, or 3 oxidized methionine residues. A peptide mass tolerance of 20 Da and MS/MS tolerance of 0.6 Da were defined.

## Results

### PDHR Identification, Functional Analysis, and Expression

**FIGURE 1:**
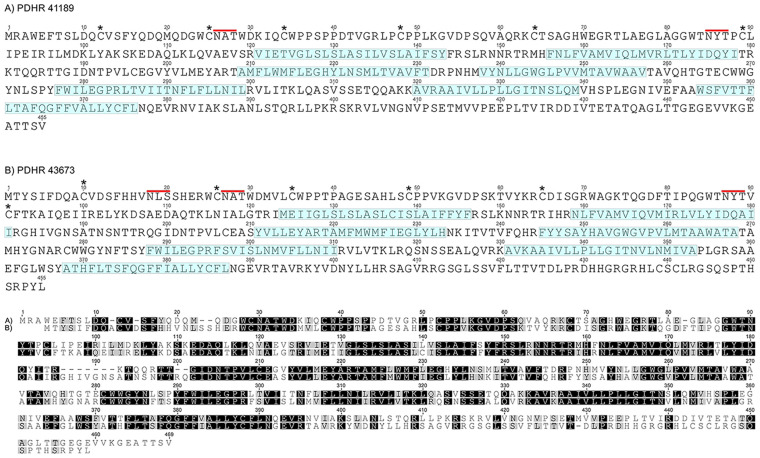
**(A)** PDHR 41189 and **(B)** PDHR 43673. Amino acid sequences of two functionally deorphaned PDH receptors. Annotations as referenced in (Oliphant et al., 2018). The six cysteine residues in the N-terminal extracellular domain are marked by asterisks, the seven predicted transmembrane regions are shaded in blue. Putative N-glycosylation sites on the extracellular N-terminal domain are indicated by red lines. Lower panel shows sequence comparisons of both receptors, highlighting identical/similar amino acids as boxed black/gray residues. Gaps added to maximize sequence identity.

**FIGURE 2:**
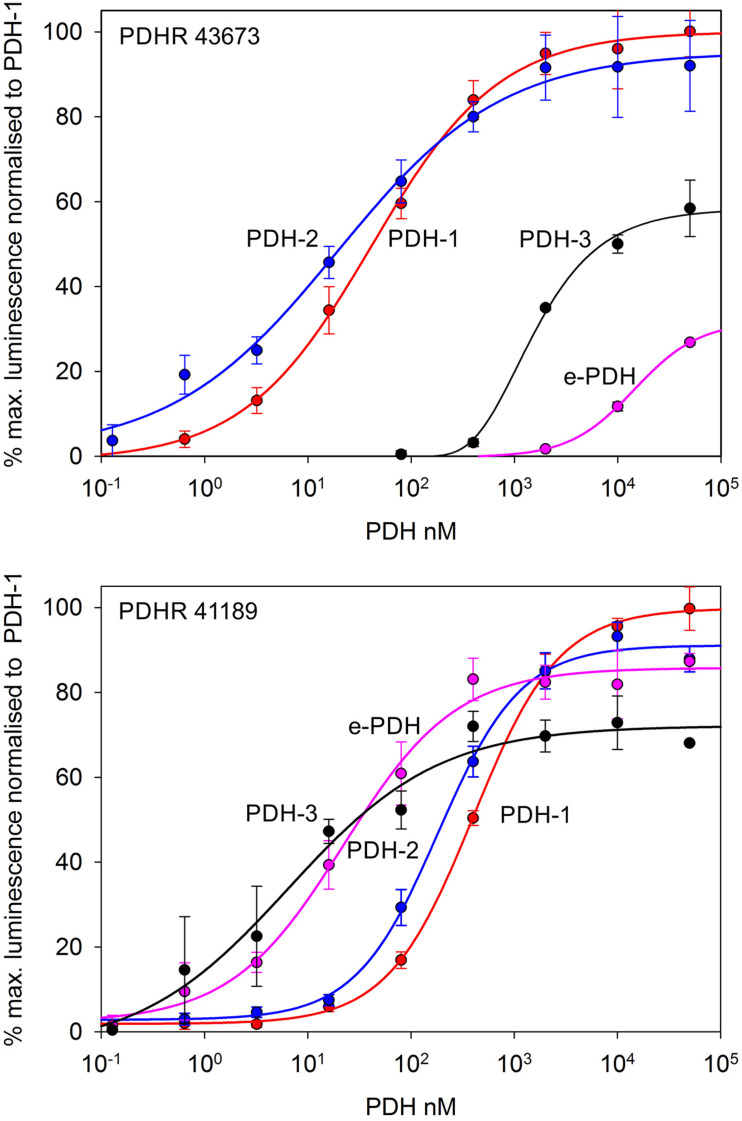
Functional confirmation of PDHR’s 43673 and 41189. Dose-response curves showing luminescence response following the addition of *C. maenas* PDH’s to CHO-K1-Aeq-Gα16 cells transiently expressing these receptor constructs. For PDHR 43673, approximate EC_50_ values were: PDH-1, 40 nM; PDH-2, 20 nM; PDH-3 >10 μM; e-PDH, >100 μM. For PDHR 41189, approximate EC_50_ values were: PDH-1, 400 nM; PDH-2, 200 nM; PDH-3, and e-PDH, 25 nM. Values are means of *N* = 4 ± 1 SD, which were normalized against the maximum luminescence responses for PDH-1.

**FIGURE 3:**
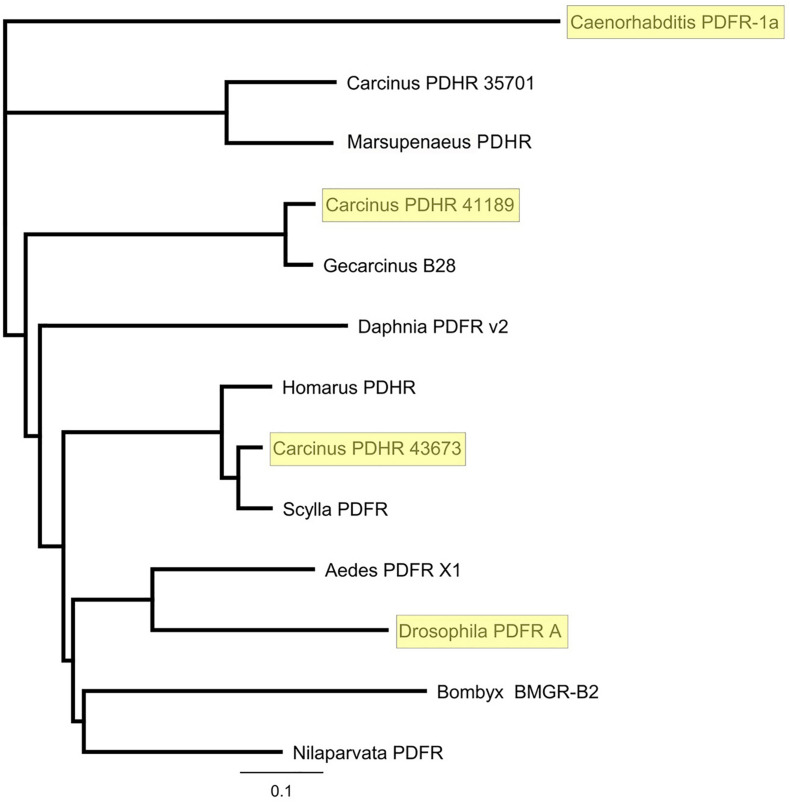
Phylogram of putative and functionally deorphaned (yellow shaded) PDHR homologs. *Carcinus maenas* PDHR 41189, 43673, and 35701 (Acc Nos: MN629919, MN629920, and MN629921, respectively); *Caenorhabditis elegans* PDFR 1a (Acc. No: EF141316), (Janssen et al., 2008); *Marsupenaeus japonicus* PDHR (Acc. No: AB478163); *Gecarcinus lateralis* B28, (Tran et al., 2019); *Daphnia pulex* PDFR v2 (Acc. No EFX90264.1) (Colbourne et al., 2011); *Homarus americanus* PDHR 14445 (Christie et al., 2015); *Scylla paramamosain* Sp-GPCR-B1, (Bao et al., 2018); *Aedes aegypti* PDFR X1 (Acc. No: XP_021711367.1) *Drosophila melanogaster* PDFR A (NP_570007.2) *Bombyx mori* BMGR-B2 (Acc. No: NP001127733.1), (Ou et al., 2014); *Nilaparvata lugens* PDFR (XP_022204430). Sequences were trimmed to include only the predicted 7-TM domains. Phylograms were assembled using Geneious V.8 Tree Builder, using a Jukes-Cantor model with the neighbor joining default setting.

Complete cDNA sequences encoding GPCRs were identified from tBLASTn searches of our *Carcinus* transcriptomes. The individual sequence read archive (SRA) codes are: SRX3280798-805, SRX3280810-814, and SRX3280830-846 and are deposited in the NCBI SRA archive as BioProject PRJNA400568. The transcriptome shotgun assembly project has been deposited at DDB/EMBL/GenBank under the accessions: GFX00000000 (Carma_CNS-transcriptome). Three full length transcripts encoding putative PDHRs were identified in our neurotranscriptomes (Transcript Nos: 35701, 41189 43673) based on identity to the *Drosophila* PDFR A and those of putative insect and crustacean PDF(H)Rs, respectively. Annotations of nucleotide and amino acid sequences of these are also available in (Oliphant et al., 2018). To avoid confusion, we have named the crustacean receptors and peptides as PDHRs and PDHs, since the peptides were originally described as circulating peptides, and are named as such in the literature, whilst those of insect and nematodes members retain their current names (PDFRs and PDFs). Of these, two (41189 and 43673) could be functionally deorphaned on the basis of their activation by *C. maenas* PDHs in the heterologous aequorin-based receptor assay, as detailed below, whilst 35701 was inactive in this respect, and thus remains an orphan. The amino acid sequences of the conceptually translated receptors are shown on Figure 1. Sequence comparison shows that both receptors have high similarity in not only the transmembrane domains, but also the (extracellular) N-termini, whilst the C-termini show very limited similarity. The 35701 sequences together with comparisons to 41189 and 43673 are shown on Supplementary Figures 1A,B. Two variants were identified from Sanger sequencing. One included a 42bp insert and both show a very high similarity to a putative PDFR from the penaeid shrimp, *Marsupenaeus japonicus*, but as alluded to earlier, these were not activated by any PDH in the receptor assay. Accession Nos are: PDHR 41189, MN629919; PDHR 43673, MN629920; PDHR-like 35701, and MN629921. Both PDHRs (ORF 1365 bp, 455 amino acids) had typical characteristics of secretin-like (Class B1) GPCRs (Prosite): Seven transmembrane domains were predicted (TMHMM 2.0 server) and likewise pfam analysis predicted a seven transmembrane interval, and 6 conserved cysteine residues in the N-terminal extracellular domain. Potential N-glycosylation sites were predicted for the N-terminal extracellular domain (NetNGlyc 1.0 server). Transient expression of both constructs into CHO-K1-Aeq cells expressing the Gα-16 subunit showed strong luminescence signals when exposed to PDHs. Control cells expressing the G*q* protein showed no response. Transfections with empty vectors showed no response. Neither receptor was activated by DH-31. The PDHRs showed striking differences in activation by the four PDHs applied as shown on Figure 2: PDHR 43673 showed much more sensitive and larger luminescence responses to PDH-1 and -2, being activated by low nanomolar concentrations of these peptides (EC_50_: PDH-1, 40 nM; PDH-2, 20 nM), whilst micromolar concentrations of PDH-3 and e-PDH were necessary to activate this receptor (EC_50_: PDH-3, 10 μM; e-PDH > 100 μM). In contrast, PDHR 41189 was activated by PDH-3 and e-PDH at low nanomolar concentrations (EC_50_: 25 nM), whereas 10 to 20-fold higher (sub-micromolar) concentrations of PDH-1 and -2 were needed to activate this receptor (EC_50_ PDH-1, 400 nM; PDH-2, 200 nM). A phylogram of selected putative and functionally deorphaned PDH and PDF receptors constructed using only the trimmed predicted 7-transmembrane domain sequences is shown on Figure 3. Inclusion of the N-terminal (extracellular) and C-terminal (intracellular) domains in this analysis had little effect on the phylogram branching.

**FIGURE 4:**
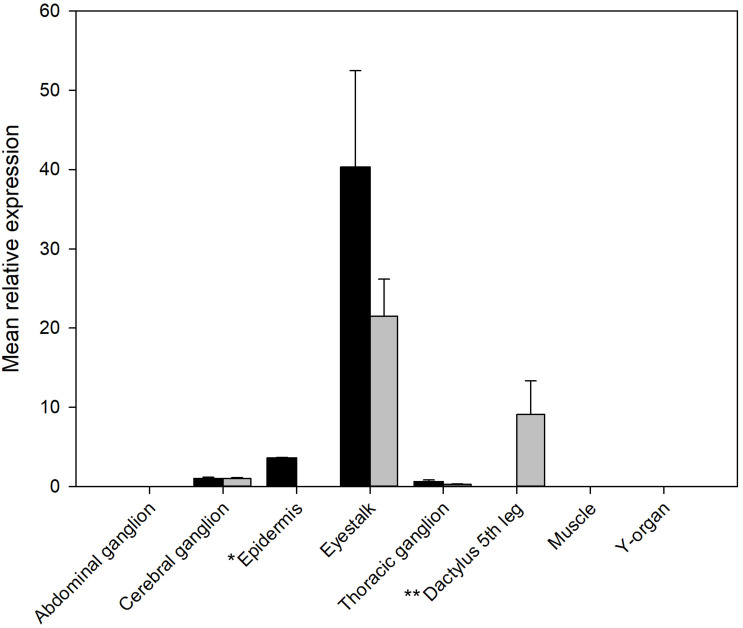
qRT-PCR showing tissue distribution and abundance of mRNA encoding PDHR 43673 (gray) and 41189 (black). *Epidermis: samples from branchiostegal region (no chromatophores), ** Dactylus: samples from muscle and epidermis chromatophores. Other tissues sampled (gill, hindgut, midgut gland, ovary, and testis) did not express detectable levels of either receptor. Values are means of *n* = 5 ± 1 SEM.

The qRT-PCR analysis of tissue distribution of both PDHRs is shown on Figure 4. Whilst both receptors were expressed at quite low levels, they showed the highest level of mRNA expression in eyestalk neural tissue, but much lower in the brain and thoracic ganglion, and for PDHR 43673 it was notable that tissues within the dactyl of the 5th walking leg (tissues from crabs of similar size to those used in bioassays), which contains large numbers of chromatophores, expressed this receptor. Epidermal tissues from areas without chromatophores (the branchiostegal region) showed a very low expression of PDHR 41189, but no detectable expression of PDHR 43673.

### PDH Sequences and Tissue Specific Expression

**FIGURE 5:**
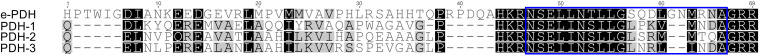
Conceptually translated amino acid sequences of *C. maenas* prepro-PDHs (excluding signal peptides), highlighting identical (black)/similar (gray) boxed amino acid residues. Gaps added to maximize sequence identity. Mature peptides are shown in blue outlined box.

**FIGURE 6:**
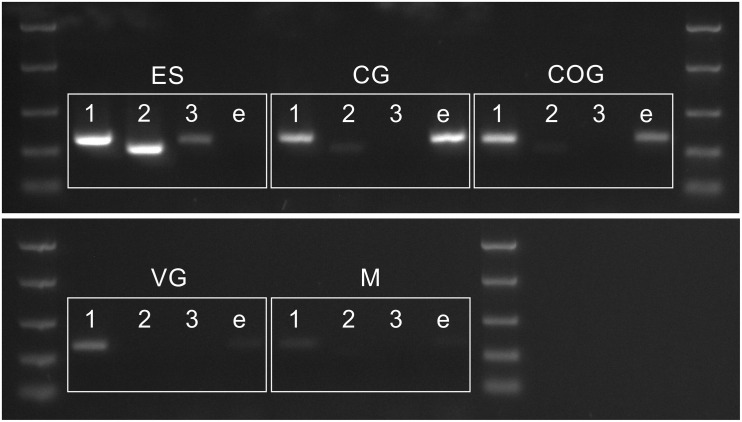
PCR showing distribution of PDH 1–3 and e-PDH transcripts in the CNS. Abbreviations: M, muscle (claw); ES, eyestalk; CG, cerebral ganglia; COG, commissural ganglia, VG ventral ganglia. DNA ladder: 2000, 1000, 500, 250, and 100 bp

**FIGURE 7:**
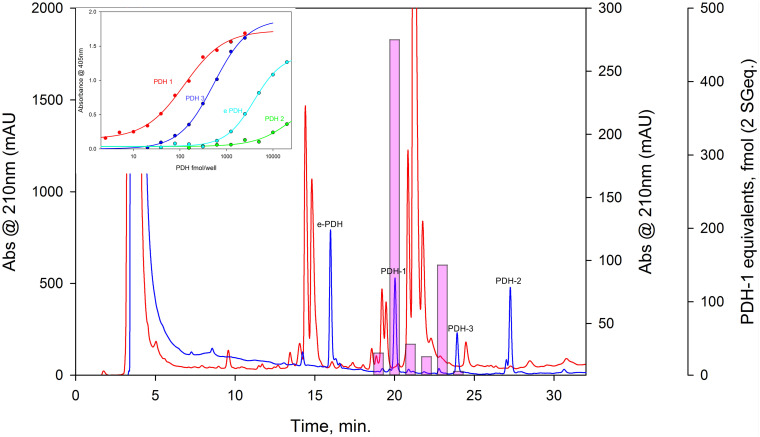
HPLC-EIA of *C. maenas* SG extracts and standard PDHs. Chromatographic conditions and EIA as described in the text. Red chromatogram shows separation of 50 SG on a Phenomenex C18 column. 1 min. fractions were assayed in duplicate for PDH immunoreactivity, shown as pink bars (PDH-1, 0.5 SG eq. per well, other PDH’s, 2.5 SG eq. per well showed no immunoreactivity). Blue chromatogram shows elution profiles of 875 pmol of each PDH, chromatographed immediately after the SG separation. Cross-reactivities of PDH’s to the PDH antiserum used (R171) are shown in the insert.

The conceptually translated sequences of four PDH-like peptides were obtained from our neurotranscriptomes; cDNAs for PDH-2,-3, ePDH, [PDH-1 has previously been fully sequenced (Klein et al., 1992)] were fully verified by 3′ and 5′ RACE, cloning and resequencing, which in addition, gave the UTR sequences. Accession Nos are: [PDH-1: L08635.1 (Klein et al., 1992)], PDH-2: MN602308, PDH-3: MN602309, e-PDH: 602310. The amino acid sequences of conceptually translated PDHs are depicted in Figure 5. The mature peptides show extensive identities in residues 1-10, i.e., some isobaric L-I substitutions (Ile^8^ vs. Leu^8^), and a Thr^7^ substitution from Ser^7^ in e-PDH. However, e-PDH contains 20 residues, compared to 18 in PDH-1-3, and there is limited sequence identity in the C-termini, excepting the C-terminal Ala-amide. The precursor-related peptides show little similarity, and for e-PDH, this is 10 residues longer (44 amino acids) than those of PDH-1-3. PCR expression of PDH in CNS tissues is shown on Figure 6. PDH-1 was expressed in all tissues (expression was very low in the ventral ganglion (VG), whilst PDH-2,-3 were only expressed in the eyestalk (ES). Expression of e-PDH was restricted to the cerebral ganglion (CG) and commissural ganglion (COG). Muscle tissue showed extremely faint PDH-1 expression, faint bands were seen for PDH-2 in the CG and COG, and e-PDH in the VG – this was perhaps due to very low levels of gDNA that remained in these preparations, since these were seen in no RT controls. Expression of PDH peptides as secretable neurohormones was determined by HPLC-EIA of SG, which showed a single immunoreactive fraction corresponding to PDH-1 (450 fmol per SG equivalent) as shown in Figure 7. However, the antiserum used showed a rather marked specificity to PDH-1 (which was originally used as immunogen), Figure 7 (insert). Immunoassay at higher concentrations (2.5 SG equivalent per well) did not reveal any immunoreactive fractions co-eluting with retention times shown for PDH-2, 3, e-PDH. LC-MS/MS of the previously HPLC separated fractions corresponding to each PDH showed fragment ions associated with PDH-1, and much weaker signals corresponding to PDH-3 which matched those annotated in the transcriptome (PDH-1: TR25293| c3_g3_il, PDH-3:TR48026| c0_g1_il). Signals corresponding to PDH-2 or e-PDH were not observed.

### PDH Expression: ISH and IHC

**FIGURE 8:**
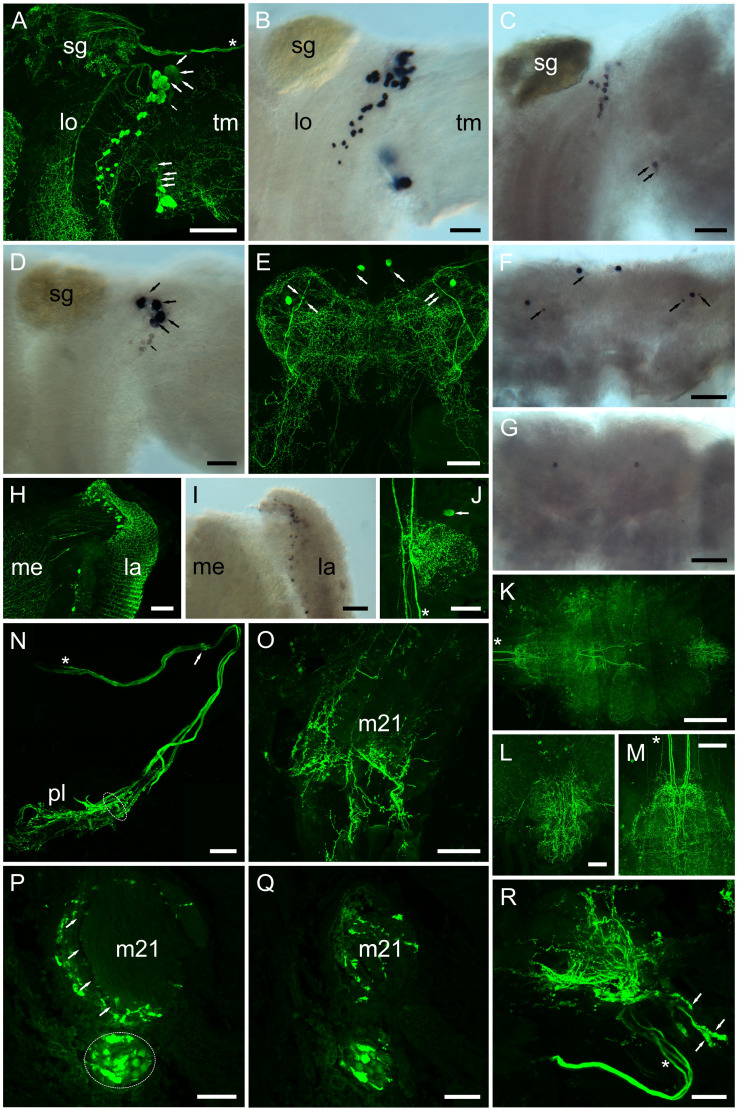
Distribution of PDHs in the central nervous system of *C. maenas*, detected by confocal ICC **(A, E, H, J–R)** or mRNA by ISH **(B–D, F, G, I)**. **(A)** Whole mount image of region between the lobula (lo) and terminal medulla (tm) of eyestalk showing intense labeling of many (*ca*. 45) PDH IR neurones and fibers. Large arrows highlight four large (40–60 μm) weakly labeled perikarya in a dorsal position adjacent to the sinus gland, and ventrally four small (*ca*. 25 μm) weakly labeled perikarya. *Small arrow* highlights a number of faintly labeled cells. Asterisk shows position of three prominent axon profiles that exit the eyestalk adjacent to the optic nerve. These profiles are also shown in **(N, R)**. **(B)** Whole mount ISH showing hybridization of PDH-1 antisense RNA to many of the profiles shown in **(A)**. **(C)** Hybridization of PDH-2 antisense probe to thirteen perikarya adjacent to the sinus gland, and two perikarya on the ventral margin of the terminal medulla (*arrows*). **(D)** Hybridization of PDH-3 antisense probe to four perikarya (*large arrows*), corresponding to the weakly labeled cells seen in **(A)**. Several (*ca*. 7) small cell bodies (*small arrows*) exhibit very weak hybridization signals. **(E)** Whole mount ICC image of the cerebral ganglion. Two pairs of intensely labeled median and lateral perikarya (*ca*. 40 μm) and weakly labeled small (*ca*. 15 μm) perikarya (arrows) are shown. **(F)** PDH-1 ISH of cerebral ganglion shows correspondence of mRNA labeling with ICC. Arrows indicate small, generally weakly hybridizing perikarya. **(G)** e-PDH ISH of cerebral ganglion showing a single pair of median perikarya. **(H)** Whole mount of lamina showing numerous small (*ca*. 10–12 μm) perikarya and fibers. **(I)** PDH-1 ISH showing hybridization signals corresponding to neuronal profiles seen in **(H)**. **(J)** Whole mount ICC image of commissural ganglion. Only one neuron was labeled (*arrow*). Asterisk indicates five descending axon profiles also indicated in **(K, M)**. **(K)**. Wholemount tiled array (5 × 4) confocal image of PDH immunopositive structures in the ventral ganglion. **(L–M)** Images of abdominal and subesophageal ganglion taken from **(K)** showing complex patterns of dendrites in both ganglia arising from thick descending axons of interneurons [*asterisks* in **(K, M)]** from within circum-esophageal connectives. **(N)** Composite confocal images showing a prominent nerve containing three stout axon profiles [*asterisk*, also indicated in **(A, R)]**, which leaves the eyestalk nervous system (exit position indicated by *small arrow*). Distally a complex branching plexus (*pl*) is seen. Dashed ellipse refers to axon bundle seen in **(P)**. **(O)** Whole mount confocal image showing branching PDH-immunopositive axons on the surface and within a small eyestalk retractor muscle (21: nomenclature according to (Burrows and Horridge, 1968). **(P)** Stacked confocal image of a 30 μm section of paraffin-embedded eyestalk showing axon profiles [dashed ellipse, as the equivalent position shown in **(N)]**. Intensely labeled putative secretory boutons are adjacent to, and within a hemolymph space (arrows). **(Q)** Confocal image of a section nearby that shown in **(P)** showing axon profiles and branching fibers throughout or alongside muscle 21. **(R)** Confocal image of a whole mount preparation similar to that shown in **(N)**. The three stout axon profiles are indicated with an *asterisk*. Structures containing putative secretory boutons are indicated by *arrows*. Abbreviations: la, lamina; lo lobula; me, medulla; m21, eyestalk retractor muscle 21; sg, sinus gland; tm, terminal medulla. Scale bars: **(K)** 500 μm; **(A, E–G)** 200 μm; **(B–D, H–, L–O, R)** 100 μm; **(P, Q)** 50 μm.

Whole mount IHC preparations using the PDH antiserum were compared to ISH preparations, where DIG – labeled antisense probes, specific for each PDH transcript were used (Figure 8) in an attempt to describe individual PDH isoform expression patterns. For eyestalk preparations, large numbers (*ca*. 45) of perikarya strongly immunoreactive (IR) to PDH were observed in the region between the terminal medulla (Tm) and lobula (Lo) (Figure 8A), and in the lamina (La). Perikarya in the former regions gave intense, positive signals for PDH-1 mRNA (Figure 8B), as well as large numbers in the lamina (Figure 8I). A smaller number of neurons (*ca.* 13) gave somewhat weaker hybridization signals to PDH-2 close to the SG, together with two weakly hybridizing cells in the anterior-ventral margin of the Tm. Four large (40–60 μm) perikarya, between the Lo and Tm, nearby the prominently labeled dorsal cell group adjacent to the SG showed very intense hybridization with the PDH-3 antisense probe (Figure 8D). These were also seen as rather faintly labeled perikarya in IHC preparations (Figure 8A, large arrows). A small number (*ca.* 7) of weakly hybridizing neurons were sometimes seen ventral to the intensely hybridizing large neurons (Figure 8D, small arrow), but were not observed in most preparations. Several small rather weakly IR cells, in the same location were also seen in IHC preparations (Figure 8A small arrow). Occasionally a single neuron was observed by IHC in the terminal medulla X-organ, but hybridization signals were never observed here. In the cerebral ganglion (CG), large (*ca* 40 μm), intensely labeled ventral – median and ventral – lateral perikarya were seen. Each of the lateral perikarya were associated with two small (*ca*. 15 μm), weakly PDH-1-IR perikarya. The two large, median perikarya were associated with a pair of small weakly labeled cells by ISH (Figure 8F). This congruence demonstrated that these cells were expressing only PDH-1. For e-PDH ISH, only a single pair of ventral – median neurons was seen in the CG, but no IR signals were detected in equivalent locations. The commissural ganglia invariably exhibited intense IR in a single neuron, but despite many attempts, ISH signals were not detected using any of the PDH probes. Five prominent descending axons entered the ventral ganglionic mass and formed very extensive branching processes (Figures 8K–M) particularly in the abdominal ganglion (Figure 8L) and even in the roots of the thoracic nerves (Figure 8K). Immunoreactive perikarya or hybridization signals were never seen in the ventral ganglionic mass, and hybridization signals were never observed using any of the PDH probes. A notable feature of the eyestalk PDH neuroanatomy was the very intensely labeling axon bundle, which exits the SG, and leaves the eyestalk neuropils mass dorsally via the protocerebral tract toward the brain proper (Figures 8A,N). This nerve contained three thick axons (shown with asterisks in Figures 8A,N,R), and in some whole mount preparations, sufficient nerve remained after dissection to show many dendrites and presumed secretory boutons (Figures 8N,R arrowheads). Thick (30 μm) paraffin wax sections were immunolabeled and images Z-stacked. These suggest that many of the presumed secretory boutons were adjacent to the prominent ophthalmic artery closely juxtaposed to a small eyestalk retractor muscle [muscle 21 according to (Burrows and Horridge, 1968)], which was also infiltrated by many small branching fibers, visible in whole mounts (Figure 8O) and sections (Figure 8Q).

### PDH Bioassay

**FIGURE 9:**
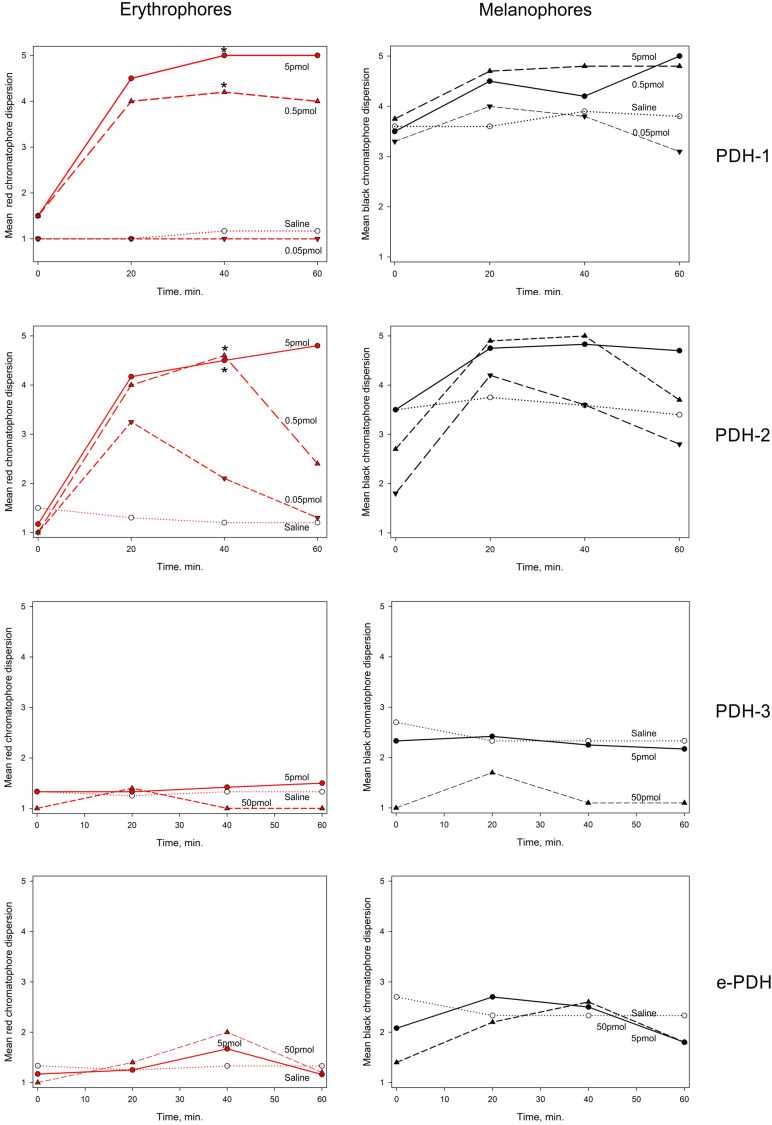
The effect of PDHs on pigment migration in epidermal chromatophores of the 5th walking leg dactylus of *C. maenas*. Left panel: erythrophores, right panel: melanophores. Peptides were injected at 0.05–5 pmol per crab for PDH-1, -2, 5–50 pmol per crab for PDH-3, e-PDH, in 10 μl volumes. Mean (*n* = 6 crabs) chromatophore indices (Hogben-Slome) are indicated. *Asterisks* indicate significant differences in chromatophore stage (*p* < 0.05, Wilcoxon sign-rank test) 40 min after injection of peptide, compared to 0 min.

Chromatophore dispersion assays as shown in Figure 9 demonstrated that both PDH-1 and -2 were effective in dispersing red chromatophores (erythrophores), with significant (*p* < 0.05, Wilcoxon-Mann Whitney rank sum test) responses to doses of around 0.5 pmol per crab 40 min after injection. For PDH-2, some pigment dispersion was observed at the lowest dose (50 fmol per crab) although this was transient and statistically not significant. These peptides also dispersed black chromatophores (melanophores), but to a lesser extent, since these were invariably partially dispersed in all crabs. PDH-3 and e-PDH were entirely without chromatophore-dispersing activity, even at the highest dose used (50 pmol per crab).

## Discussion

By using a reverse pharmacology approach, we have identified two PDH receptor isoforms (PDHR41189, 43673) in the green shore crab, *C. maenas* which can be activated by a surprisingly large inventory (4 isoforms) of crustacean PDHs in this species. These are the first PDH receptors to be deorphaned in any crustacean; indeed, to date whilst a large variety of neuropeptide/receptor pairings have been tentatively identified in crustaceans from *de novo* transcriptome assembly mining, or genome annotations, [for example (Christie et al., 2013, 2017; Bao et al., 2018; Tran et al., 2019)] to our knowledge, only a few receptor/ligand pairings have been functionally deorphaned, using cell-based heterologous reporter assays. These include the red pigment concentrating hormone receptor (RPCHR) in *Daphnia pulex* (Marco et al., 2017), RPCHR, corazonin receptor (CRZR) and diuretic hormone 31 receptor (DH31R) in *C. maenas* (Alexander J. et al., 2018; Alexander J. L. et al., 2018) and crustacean cardioactive peptide receptor (CCAPR) in *Scylla paramamosain* (Bao et al., 2018). With regard to PDF/PDHs and their receptors, we were particularly intrigued by the diversity of diversity of peptides and receptors in *C. maenas*, since insects seem to have only single PDF peptides and putative PDFRs annotated in databases, yet other non-insect ecdysozoans such as nematodes, *viz*. *Caenorhabditis elegans* have multiple isoforms of both PDFs and PDFRs (Janssen et al., 2008, 2009). An emerging scenario in decapod crustaceans is that they possess multiple PDHs, with two or three PDHs commonly found [e.g., (Klein et al., 1994; Ohira et al., 2002; Bulau et al., 2004; Hsu et al., 2008; Huang et al., 2014)]. Our finding that *C. maenas* possesses three isoforms of the conventional 18 amino acid β-PDH and a 20 residue C-terminally extended peptide (e-PDH) reinforces this view, and it seems likely that the extended PDH isoform is common to all decapod crustaceans (Veenstra, 2016).

### PDHRs: Structures Activities and Specificities

The PDHRs (43673 and 41189) that were activated by PDH showed very marked selectivity with regard to dose; PDH-1 and -2 activated the 43673 receptor in the low nanomolar range, whereas even micromolar concentrations of PDH-3 and e-PDH were needed to produce rather weak luminescence responses. This situation was reversed for the 41189 receptor, where low nanomolar concentrations of PDH-3 and e-PDH activated this receptor, but high nanomolar concentrations of PDH-1, -2 were needed to result in a luminescent signal. Whilst it is likely that the biological activity of the PDHs in the cell-based assay are rather different to those *in vivo*, if an assumption is made that they may be in the same order of magnitude, or at least related, the highest doses of PDH 1-2 would (assuming a hemolymph volume of 100 μl for a 15–20 mm carapace width crab) give an instantaneous hormone concentration of around 50 nM – e.g., well within the concentration range needed to activate the 43673 receptor and evoke a biological response – i.e., chromatophore dispersion *in vivo*. For PDH-3 and e-PDH this concentration would be insufficient to activate the receptor or to result in pigment dispersion *in vivo*. However, for receptor 41189, the converse could be true. This implicates the 43673 receptor in chromatophore dispersion. In *Drosophila*, DH31 activates the PDF receptor to some extent (Mertens et al., 2005), and it has recently been established that PDF and DH31 hierarchically regulate free-running rhythmicity in male flies, and that *dh31* and *pdf* double mutants exhibit a severely disrupted arrhythmic phenotype compared to *pdf* null mutants alone (Goda et al., 2019). In our heterologous assay, we could not activate any of the PDH receptor with DH31 even at μM concentrations. Nevertheless, it would be interesting to speculate, and investigate whether DH31, or its cognate receptor are expressed in PDH neurones, or *vice versa*, which might shed light on commonalities of clock mechanisms between crustaceans and insects.

A phylogram of selected PDF receptors for insects, compared with crustaceans, and a deorphaned PDFR from *C. elegans*, which forms the outgroup, as expected, clearly showed that 43673 groups with the crab *Scylla paramamosain*, and lobster *Homarus americanus*, and forms an outgroup to the insect PDFRs, yet the other functionally deorphaned PDH receptor, 41189, was more closely related to that of the land crab, *Gecarcinus lateralis.* This situation is interesting, since the *G. lateralis* receptor was annotated from Y-organ transcriptomes (Tran et al., 2019). It has been reported that insect PDF regulates (stimulates) ecdysone biosynthesis in the prothoracic glands (PGs) of the silkworm *Bombyx mori* and that this occurs via the neuropeptide GPCR-B2, which is annotated as a PDFR (Iga et al., 2014). This stimulation, which is only found in post gut-purged larvae and pupae, was distinct from that of prothoracicotropic hormone (PTTH) and involved both cAMP and Ca^2+^ mediated pathways. Surprisingly, we have also found the PDH receptor 41189 (but not that of 43673) in our YO transcriptomes [transcript 34919, (Oliphant et al., 2018)]. However, it should be noted that this transcript, although identical in sequence, was a little incomplete and N-terminally truncated (8 amino acids). However, this transcript could not be detected by PCR of carefully dissected YO, thus we assume that this may reflect tissue contamination, possibly due to epidermal remnants.

The qRT-PCR of RNA from a wide variety of tissues showed quite low levels of expression of both PDHRs, primarily in eyestalk tissues. However, it was notable that PDHR 43673 expression was found in tissue from the dactyl of the fifth walking leg of juvenile crabs, which contains large numbers of chromatophores in the epidermal tissues. Epidermis samples that did not contain chromatophores, taken from the branchiostegal region expressed low levels of PDHR 41189. This result suggests that PDHR 43673 is likely the authentic PDH neurohormone receptor, which is in accordance with its pronounced selectivity for PDH-1, -2, as these PDH-isoforms exhibit biological activity in the chromatophore dispersion assay, making this hypothesis clearly a very an attractive one. The third putative PDHR (35701) (Oliphant et al., 2018) could not be activated by any of the four PDHs and showed rather limited similarity to the other PDHRs (43673,41189) but had a very high sequence identity to a GPCR annotated as a PDHR in the penaeid shrimp *Marsupenaeus japonicus* (Acc. No. AB478163). This highlights the issue of functional deorphaning being a necessary prerequisite before annotations become entrenched in the literature (Caers et al., 2012).

### Differential Localizations of PDHs and Their PDHRs

With regard to involvement of PDH (receptor-mediated) signaling in crustacean circadian clockwork, the finding that a homolog of PDHR 43673 is expressed in brain and eyestalk transcriptomes of the lobster *Homarus americanus* (Christie et al., 2017) is interesting. Both PDHRs (41189 and 43673) were expressed in the eyestalk, cerebral and thoracic ganglia of *C. maenas* and could be candidates for components of the circadian clockwork. Whilst many reports suggest that cardiac output shows features of endogenous rhythmicity *in vivo* and *in vitro* [references in (Christie et al., 2018)], transcriptomic analysis of cardiac ganglion tissues of *Homarus americanus* (which contain pacemaker centers) did not show expression of a PDHR, whilst expressing a wide variety of canonical clock genes which could form the basis of a peripheral circadian oscillator (Christie et al., 2018).

Our findings on the selectivity and distributions of the two PDHRs clearly suggest distinct functions for the various PDHs. Thus, we attempted to identify the expression of the various PDH isoforms in the CNS. PCR showed that PDH-1 mRNA had ubiquitous expression throughout the CNS, whilst the distribution of the others was somewhat limited; PDH-2 and -3 were only found in eyestalk tissues, whilst e-PDH was strongly expressed in the cerebral ganglion and to a lesser extent the commissural ganglion. ISH confirmed this expression profile (although hybridizing perikarya were not seen in the COG or VG). For PDH-1, large numbers of hybridizing neurons were seen in the eyestalk, near the lobula – medulla boundary and in the lamina. These mapped to peptide expression patterns using an antiserum raised against *C. maenas* PDH-1, which showed a marked selectivity for this peptide. The neuronal architecture (positions of neurons, tracts etc.) obtained in this study are in broad agreement (but not identical) with immunoreactive structures in the CNS of *C. maenas* previously described in detail using another anti-PDH serum raised against an identical PDH-1 (Mangerich et al., 1987; Mangerich and Keller, 1988) and also for the CNS of *Cancer productus* (Hsu et al., 2008), excepting the absence of IR and hybridizing perikarya in the ventral ganglion. In the latter study, double ISH experiments and matrix-assisted laser desorption/ionization Fourier transform mass spectrometry (FT-MS) showed most neurons in the region between the lobula and medulla expressed both a PDH-1 that was identical in sequence to *C. maenas* PDH-1 and a second PDH structurally similar, but not identical, to the one identified in this study (PDH-2). However, a few expressed only the PDH-2 isoform, and the amacrine neurons in the lamina only expressed PDH-1. In the present study, only a small set of neurons (13) adjacent to the SG, and two anterior-ventral ones in the terminal medulla expressed PDH-2. As alluded to above, the anti-PDH serum used was raised to PDH-1, and showed marked selectivity (doses required to give an absorbance of 1.0 in the EIA were: PDH-1 150 fmol; PDH-2 ≫10,000 fmol, PDH-3 600 fmol; e-PDH 8000 fmol, see Figure 7 inset), therefore it seems unlikely that the PDH-2 peptide could be detected, but it is clear that the IR neurons in the lamina only express PDH-1 as also observed for *C. productus* (Hsu et al., 2008) and implicates a similar existence of PDH-1 in lamina amacrine neurons of *C. maenas* has already been shown much earlier (Mangerich et al., 1987). PDH-3 mRNA expression was restricted to four large (ca. 50 μm), intensely hybridizing large perikarya and several very weakly hybridizing ones (that were not seen in most preparations). Since the PDH antiserum showed reasonable cross reactivity (about 25%) to this PDH isoform it is tempting to suggest that these four large, weakly immunoreactive cells in the prominent group of neurons adjacent to the SG, and the smaller ones nearby, represent PDH-3 expressing neurons.

For the cerebral ganglion the ventral – median and ventral – lateral neurons seen in IHC, mapped precisely with the ISH signals for the PDH-1 probe. Thus, we can be certain that these neurons only express PDH-1. Such PDH-neurons of the CG would merit investigation for their contributions to long-known activities in circadian rhythmicity control driven by histaminergic brain photoreceptors that contact PDH-neurons in the CG of other decapods (Sullivan et al., 2009; Strauss and Dircksen, 2010). A further pair of ventral – median cells expressing e-PDH was observed in the cerebral ganglion. These neurons invariably gave quite weak hybridization signals, as opposed to the robust amplification of e-PDH in PCR. These were visible in all preparations, but due to the selectivity of the antiserum, these perikarya were not seen in any IHC preparations. Whilst both PDH-1 and e-PDH gave clear amplification signals in PCR of the commissural ganglia (Figure 6) hybridizing neurons were never observed, despite the presence of intense immunoreactivity in one neuron, which was likely that of PDH-1. Given the undeniably complex anatomy of PDH neurons, together with our observations on the variety of PDHs and their corresponding neuronal mRNA expression, it is now timely to describe the detailed neuroanatomy for each peptide. This is eminently feasible, since the four PDH-related peptide sequences of *C. maenas* are probably sufficiently individually distinctive to raise precursor-specific antisera, whereas it is likely that the sequence similarity of the mature PDH peptides would probably preclude this.

A pertinent question now arises: how can we differentiate between PDHs with neurohormonal vs. those with neurotransmitter/modulator roles? Clearly, PDH-1 has a neurohormonal role, since it is long known as being released from a principle secretory tissue, the sinus gland, as evidenced by HPLC-EIA and MS, yet it also has transmitter/modulatory roles, as shown by the presence of IR interneurons and arborizing dendrites in the lamina, cerebral ganglia and very complex processes throughout the ventral ganglion, as previously noted (Mangerich et al., 1987; Mangerich and Keller, 1988). However, in *C. productus* only PDH-2 was found in the sinus gland (Hsu et al., 2008), leading these authors to suggest a purely neurohormonal role for this peptide. In *C. maenas*, as alluded to earlier, PDH-2 bears significant sequence similarities, and is undoubtedly a homolog to the *C. productus* peptide, and also that of *C. sapidus* (Klein et al., 1994) and *S. paramamosain* (Huang et al., 2014), whilst PDH-1 is identical in all these species. Characterization of the *C. maenas* neuropeptide inventory by MALDI-TOF MS and FT-MS has previously shown that only PDH-1 is found in the SG (Ma et al., 2009), but in that study the occurrence of PDH isoforms in other tissues of the CNS was not performed. Our previous identification of PDH-1 in *C. maenas* (Löhr et al., 1993) by immunoaffinity purification of eyestalks (including SG), followed by HPLC showed several PDH immunoreactive fractions, but a prominent peak corresponding to PDH-1 by retention time was sequence-confirmed by FAB-MS. Our MS studies reported here showed that PDH-2 is not found in the SG, so this clearly does not point to a neurohormonal role, unless other release phenomena could come into play, as have been proposed by electron microscopic demonstration of putative intra-neuropilar release sites for peptides in a crayfish central body (Schürmann et al., 1991). Nevertheless, PDH-2 was very potent in the chromatophore dispersion assay. However, in *C. sapidus*, PDH-2 is at least 400-fold less active than PDH-1, in dispersing chromatophores (Klein et al., 1994), which is at odds with our findings for *C. maenas*. However, since PDH-2 does not appear to have a neurohormonal role in the latter crab, on the basis of absence in the SG in this study and (Ma et al., 2009), its pigment dispersing activity *in vivo* must be due to promiscuous binding and activation of the PDH (43673) receptor. Since PDH-3 was found in HPLC separated SG by LC-MS (but not HPLC EIA), it is tempting to suggest that this peptide might have a neurohormonal role, but this must be unrelated to pigment dispersion, since it is entirely without pigment dispersing activity in the chromatophore bioassay. A likely explanation for the possible localization of PDH-3 in the SG could be that during dissection, the PDH-3 expressing cells and terminals adjacent to but outside the SG were inadvertently included as contaminants.

*Carcinus maenas* juveniles exhibit a robust endogenous circadian rhythm of red and black chromatophore dispersion during times of expected photophase, and concentration at times of expected scotophase, which persists under constant darkness for over 35 days, but without a circatidal (12.4 h) component (Powell, 1962). Thus, release of PDH-1 from the sinus gland (and the newly described putative neurohemal tissue associated with the eyestalk retractor muscle 21 described here) certainly reflect their roles as key neurohormonal outputs of the circadian clock, apart from any neurotransmitter/modulator functions.

Since PDH-1 profiles were quite sparse in the SG, and PDH IR axons leave the eyestalk neural tissues adjacent to the optic nerve in *C. maenas, Orconectes limosus*, (Dircksen et al., 1987; Mangerich et al., 1987; Mangerich and Keller, 1988; Dircksen, 1992) and *C. productus* (Hsu et al., 2008), we were interested in tracing this nerve, which contained three very thick, intensely labeled axons. In a few whole mount preparations of eyestalk neural tissues, 1–1.5 mm sections of this nerve, external to the eyestalk neural mass were coincidentally dissected (Figures 8N,R), and arborizations reminiscent of neurosecretory structures were seen. Sections of complete eyestalks, including muscles showed these arborizations were not only directly adjacent to an artery on an eyestalk muscle, but that many fine fibers were also present within this muscle (Figure 8Q), as also seen in coincidentally dissected eyestalk muscles in whole mount preparations (Figure 8O). This muscle was identified as muscle 21 of the eyecup of *C. maenas*, one of the nine involved in optokinetic movements, and eyestalk retraction (Burrows and Horridge, 1968). Although, ultrastructural investigation of the presumed neurosecretory nature of the boutons adjacent to the (ophthalmic) artery has yet to be done, it is obvious that this region contains much more PDH than that in the SG. Since the originally described function of PDH was in distal retinal pigment migration, where ommatidia are protected from bright daylight conditions, it is tempting to speculate that the PDH fibers in muscle 21 may be involved in eyestalk retraction into the eyecup to further protect the photoreceptors. In this context, it is interesting to note that in barnacles, the somatic extensor muscles (the attrahens muscle) are innervated via the great splanchnic nerve and finely branching PDH IR fibers and secretory boutons are extensively distributed over these muscles (Webster, 1998), in a manner reminiscent of that seen for eyestalk retractor muscle 21. Thus, it seems possible that PDH has a relevant function in modulation of muscle contractility, possibly in response to bright light conditions. It would be interesting to test this hypothesis by quantifying behaviors, for example optokinetic movements, in light and dark conditions, and after PDH-1 injection.

## Conclusion

This study details the characterization of PDH signaling in a crustacean for the first time. We demonstrate that four pigment dispersing-type hormones preferentially activate two distinct receptors. The signaling system associated with PDHR 43673 that involves PDH-1 is likely to represent that of the neurohormonal pathway leading to chromactivation, notwithstanding other functions in neuromodulation. Additionally, neuroanatomical studies revealed a previously undescribed neurohemal area and secretory boutons in one of the eyestalk retractor muscles, which is likely to be involved in photic adaptation. The signaling system involving the receptor PDHR 41189 is preferentially activated by PDH-3 and e-PDH; these peptides are expressed by interneurons with functions in neuromodulation. To further define the roles of these peptides and receptors in fundamental processes in regulation of rhythmic locomotor and chromatic adaptation behavior, it is now timely to further define the roles of specific PDH ligands and receptors, by developing knockdown experiments, together with phenotypic observations associated with these functions.

## Data Availability Statement

The datasets presented in this study can be found in online repositories. The names of the repository/repositories and accession number(s) can be found in the article/Supplementary Material.

## Ethics Statement

This work involved animals (crustaceans). Crustaceans are not covered by the United Kingdom Home Office Animal Scientific Procedures Act 1986 or Directive 2010/63/EU, but we adhered to our local ethical guidelines, minimized the number of animals used as far as possible, and ice-anesthetized animals prior to dissections. *C. maenas* were collected from the Menai Strait using baited traps. Collection of *C. maenas* from this location is not restricted or subject to local or national licensing or permissions.

## Author Contributions

JA, SW, and DW designed the research. JA, AO, SW, DW, and TB-S performed the research (JA and AO contributed equally). SW, DW, and HD wrote the manuscript. All authors contributed to the article and approved the submitted version.

## Conflict of Interest

The authors declare that the research was conducted in the absence of any commercial or financial relationships that could be construed as a potential conflict of interest.

## Supplementary Material

Supplementary Figure 1 **(A)** Amino acid alignments *C. maenas* PDHR-like 35701 and *Marsupenaeus japonicus* AB778163. Amino acid alignment of *C. maenas* 35701, from transcriptome, and clones identified by PCR and Sanger sequencing, compared to a putative PDHR from *Marsupenaeus japonicus* (AB 478163) highlighting identical (black)/similar (gray) boxed amino acid residues. Gaps added to maximize sequence identity. The seven predicted transmembrane domains are highlighted in blue, six conserved extracellular N-terminal domain cysteines are marked by asterisks, and putative N-glycosylation sites on the extracellular N-terminal domain are indicated by red lines. **(B)** Amino acid alignments *C. maenas* PDHR-like 35701, PDHR 41189, and 43673

Supplementary Table 1 Primers used.

## Abbreviations

ABTS: 2,2 ′ -Azino-bis(3-ethylbenz-thiazoline-6-sulfonic acid) diammonium salt
BSA: bovine serum albumen
cAMP: cyclic adenosine monophosphate
CCAPR: crustacean cardioactive peptide receptor
cDNA: complementary DNA
CG: cerebral ganglion
CHO: chinese hamster ovary
CNS: central nervous system
COG: commissural ganglion
CRZR: corazonin receptor
DH-31: diuretic hormone-31
DH31R: diuretic hormone-31 receptor
DIG: digoxygenin
DMEM/F: Dulbecco’s Modified Eagle Medium F-12
EIA: enzyme immunoassay
ERG: electroretinogram
ES: eyestalk
FAB-MS: fast atom bombardment-mass spectrometry
FT-MS: fourier transform-mass spectrometry
gDNA: genomic DNA
GPCR: G protein-coupled receptor
HEPES: N-(2-Hydroxyethyl)piperazine-N’-(2-ethanesulfonic acid)
H-ESI: heated electrospray ionization
HPLC: high performance liquid chromatography
IHC: immunohistochemistry
IR: immunoreactive
ISH: *In situ* hybridization
La: Lamina
LC MS/MS: liquid chromatography tandem mass spectrometry
Lo: lobula
MALDI-TOF MS: matrix-assisted laser desorption/ionization-time of flight mass spectrometry
Me: medulla
mRNA: messenger RNA
Mt: terminal medulla
ORF: open reading frame
PCR: polymerase chain reaction
PDF: pigment dispersing factor
PDH: pigment dispersing hormone
PDFR: pigment dispersing factor receptor
PDHR: pigment dispersing hormone receptor
PBS: phosphate buffered saline
PBST, PBS: 0.1% Tween 20
PFA: paraformaldehyde
PTTH: prothoracicotropic hormone
qRT-PCR: quantitative reverse transcriptase polymerase chain reaction
RLM RACE: RNA ligase-mediated rapid amplification of cDNA ends
RPCHR: red pigment concentrating hormone receptor
SRA: sequence read archive
SG: sinus gland
TFA: trifluoroacetic acid
UHPLC: Ultra-HPLC
UTR: untranslated region
VG: ventral ganglion
VPAC2: mammalian vasoactive intestinal peptide receptor.

## References

[B1] Alexander J. Oliphant A. Wilcockson D. C. Webster S. G. (2018). Functional identification of the diuretic hormone 31 (DH31) signalling system in the green shore crab, *Carcinus maenas*. *Front. Neurosci.* 12:454. 10.3389/fnins.2018.00454 30022930 PMC6039563

[B2] Alexander J. L. Oliphant A. Wilcockson D. C. Audsley N. Down R. E. Lafont R. (2018). Functional characterization and signaling systems of corazonin and red pigment concentrating hormone in the green shore crab, *Carcinus maenas*. *Front. Neurosci.* 11:752. 10.3389/fnins.2017.00752 29379412 PMC5775280

[B3] Aréchiga H. Fernández-Quiróz F. de Miguel F. F. Rodríguez-Soza L. (1993). The circadian system of crustaceans. *Chronobiol. Int.* 10 1–19. 10.3109/07420529309064477 8443839

[B4] Aréchiga H. Rodríguez-Soza L. (1998). Circadian clock function in isolated eyestalk tissue of the crayfish. *Proc. R. Soc. Lond. B* 265 1819–1823. 10.1098/rspb.1998.0507 9802237 PMC1689369

[B5] Artimo P. Jonnalagedda M. Arnold K. Baratin D. Csardi G. de Castro E. (2012). ExPASy: SIB bioinformatics resource portal. *Nucleic Acids Res.* 40 W597–W603. 10.1093/nar/gks400 22661580 PMC3394269

[B6] Bao C. Yang Y. Zeng C. Huang H. Ye H. (2018). Identifying neuropeptide GPCRs in the mud crab, *Scylla paramamosain*, by combinatorial bioinformatics analysis. *Gen. Comp. Endocrinol.* 269 122–130. 10.1016/j.ygcen.2018.09.002 30189191

[B7] Bulau P. Meisen I. Schmitz T. Keller R. Peter-Katalinić J. (2004). Identification of neuropeptides from the sinus gland of the crayfish *Orconectes limosus* using nanoscale on-line liquid chromatography tandem mass spectrometry. *Mol. Cell. Proteomics* 3 558–564. 10.1074/mcp.M300076-MCP200 14981133

[B8] Burrows M. Horridge G. A. (1968). The action of the eyecup muscles of the crab, *Carcinus*, during optokinetic movements. *J. Exp. Biol.* 49 223–250.

[B9] Caers J. Verlinden H. Zels S. Vandermissen H. P. Vuerinckx K. Schoofs L. (2012). More than two decades of research on insect neuropeptide GPCRs: an overview. *Front. Endocrinol.* 3:151. 10.3389/fendo.2012.00151 23226142 PMC3510462

[B10] Christie A. E. Chi M. Lameyer T. J. Pascual M. G. Shea D. N. Stanhope M. E. (2015). Neuropeptidergic signalling in the American lobster *Homarus americanus*: new insights from high throughput nucleotide sequencing. *PLoS One* 10:e145964. 10.1371/journal.pone.0145964 26716450 PMC4696782

[B11] Christie A. E. Nolan D. H. Garcia Z. A. McCoole M. D. Harmon S. M. Congdon-Jones B. (2011). Bioinformatic prediction of arthropod/nematode-like peptides in non-arthropod, non-nematode members of the Ecdysozoa. *Gen. Comp. Endocrinol.* 170 480–486. 10.1016/j.ygcen.2010.11.002 21074533

[B12] Christie A. E. Roncalli V. Cieslak M. C. Pascual M. G. Yu A. Lameyer T. J. (2017). Prediction of a neuropeptidome for the eyestalk ganglia of the Lobster *Homarus americanus* using a tissue specific *de novo* assembled transcriptome. *Gen. Comp. Endocrinol.* 243 96–119. 10.1016/j.ygcen.2016.11.001 27823957 PMC5796769

[B13] Christie A. E. Roncalli V. Wu L. S. Ganote C. L. Doak T. Lenz P. (2013). Peptidergic signaling in *Calanus finmarchicus* (Crustacea, Copepoda): *In silico* identification of putative peptide hormones and their receptors using a *de novo* assembled transcriptome. *Gen. Comp. Endocrinol.* 187 117–135. 10.1016/j.ygcen.2013.03.018 23578900

[B14] Christie A. E. Yu A. Roncalli V. Pascual M. G. Cieslak M. C. Warner A. N. (2018). Molecular evidence for an intrinsic circadian pacemaker in the cardiac ganglion of the American lobster, *Homarus americanus*-Is diel cycling of heartbeat frequency controlled by a peripheral clock system? *Mar. Genomics* 41 19–30. 10.1016/j.margen.2018.07.001 30031746

[B15] Colbourne J. K. Pfrender M. E. Gilbert D. Thomas W. K. Tucker A. Oakley T. H. (2011). The ecoresponsive genome of *Daphnia pulex*. *Science* 331 555–561. 10.1126/science.1197761 21292972 PMC3529199

[B16] Dircksen H. (1992). Fine structure of the neurohemal sinus gland of the shore crab, *Carcinus maenas*, and immuno-electron-microscopic identification of neurosecretory endings according to their neuropeptide contents. *Cell Tiss. Res.* 269 249–266. 10.1007/BF00319616 1423493

[B17] Dircksen H. Zahnow C. A. Gaus G. Keller R. Rao K. R. Riehm J. P. (1987). The ultrastructure of nerve endings containing pigment-dispersing hormone (PDH) in crustacean sinus glands: identification by an antiserum against a synthetic PDH. *Cell Tiss. Res.* 250 377–387. 10.1007/BF00219082

[B18] Elekes K. Nässel D. R. (1999). Pigment-dispersing hormone-like immunoreactive neurons in the central nervous system of the gastropods, *Helix pomatia* and *Lymnaea stagnalis*. *Cell. Tiss. Res*. 295 339–348. 10.1007/s004410051240 9931380

[B19] Elphick M. R. Mirabeau O. Larhammar D. (2018). Evolution of neuropeptide signalling systems. *J. Exp. Biol.* 221:jeb151092. 10.1242/jeb.193342 29440283 PMC5818035

[B20] Fernlund P. (1976). Structure of a light-adapting hormone from the shrimp, *Pandalus borealis*. *Biochem. Biophys. Acta* 439 17–25. 10.1016/0005-2795(76)90155-0 952951

[B21] Goda T. Umezaki Y. Alwattari F. Seo H. W. Hamada F. N. (2019). Neuropeptides PDF and DH31 hierarchically regulate free-running rhythmicity in *Drosophila* circadian locomotor activity. *Sci. Rep.* 9:938. 10.1038/s41598-018-37107-3 30696873 PMC6351594

[B22] Helfrich-Förster C. (2009). Neuropeptide PDF plays multiple roles in the circadian clock of *Drosophila melanogaster*. *Sleep Biol. Rhythms* 7 130–143. 10.1111/j.1479-8425.2009.00408.x

[B23] Helfrich-Förster C. Tauber M. Park J. H. Muhlig-Versen M. Schneuwly S. Hofbauer A. (2000). Ectopic expression of the neuropeptide pigment-dispersing factor alters behavioral rhythms in *Drosophila melanogaster*. *J. Neurosci.* 20 3339–3353. 10.1523/JNEUROSCI.20-09-03339.2000 10777797 PMC6773135

[B24] Hoelters L. O’Grady J. F. Webster S. G. Wilcockson D. C. (2016). Characterization, localization and temporal expression of crustacean hyperglycemic hormone (CHH) in the behaviourally rhythmic peracarid crustaceans, *Eurydice pulchra* (Leach) and *Talitrus saltator* (Montagu). *Gen. Comp. Endocrinol.* 237 43–52. 10.1016/j.ygcen.2016.07.024 27468954

[B25] Hogben L. Slome D. (1931). The pigmentary effector system VI. The dual character of endocrine co-ordination in amphibian colour change. *Proc. Roy. Soc. Lond. B* 108 10–53. 10.1098/rspb.1931.0020

[B26] Hsu Y.-W. A. Stemmler E. A. Messinger D. I Dickinson P. S. Christie A. E. de la Iglesia H. O. (2008). Cloning and differential expression of two β-pigment-dispersing hormone (β-PDH) isoforms in the crab *Cancer productus*: evidence for authentic β-PDH as a local neurotransmitter and β-PDH II as a humoral factor. *J. Comp. Neurol.* 508 197–211. 10.1002/cne.21659 18311785

[B27] Huang X. Ye H. Huang H. Yu K. Huang Y. (2014). Two beta-pigment dispersing hormone (β-PDH) isoforms in the mud crab, *Scylla paramamosain*: implication for regulation of ovarian maturation and a photoperiod-related daily rhythmicity. *Anim. Reprod. Sci.* 150 139–147. 10.1016/j.anireprosci.2014.09.004 25262380

[B28] Hyun S. Lee Y. Hong S. T. Bang S. Paik D. Kang J. (2005). *Drosophila* GPCR Han is a receptor for the circadian clock neuropeptide PDF. *Neuron* 48 267–278. 10.1016/j.neuron.2005.08.025 16242407

[B29] Iga M. Nakaoka T. Suzuki Y. Katakoa H. (2014). Pigment dispersing factor regulates ecdysone biosynthesis via *Bombyx* neuropeptide G protein coupled receptor-B2 in the prothoracic glands of *Bombyx mori*. *PLoS One* 9:e103239. 10.1371/journal.pone.0103239 25072638 PMC4114559

[B30] Janssen T. Husson S. J. Lindemans M. Mertens I. Rademakers S. Ver Donck K. (2008). Functional characterization of three G protein-coupled receptors for pigment dispersing factors in *Caenorhabditis elegans*. *J. Biol. Chem.* 283 15241–15249. 10.1074/jbc.M709060200 18390545 PMC3258896

[B31] Janssen T. Husson S. J. Meelkop E. Temmerman L. Lindemans M. Verstraelen K. (2009). Discovery and characterization of a conserved pigment dispersing hormone-like neuropeptide pathway in *Caenorhabditis elegans*. *J. Neurochem.* 111 228–241. 10.1111/j.1471-4159.2009.06323.x 19686386

[B32] Jekely G. (2013). Global view of the evolution and diversity of metazoan neuropeptide signaling. *Proc. Natl. Acad. Sci. U.S.A.* 110 8702–8707. 10.1073/pnas.1221833110 23637342 PMC3666674

[B33] Klein J. M. de Kleijn D. P. Keller R. Weidemann W. M. (1992). Molecular cloning of crustacean pigment dispersing hormone precursor. *Biochem. Biophys. Res. Commun.* 189 1509–1514. 10.1016/0006-291X(92)90246-H 1282803

[B34] Klein J. M. Mohrherr C. J. Sleutels F. Riehm J. P. Rao K. R. (1994). Molecular cloning of two pigment-dispersing hormone (PDH) precursors in the blue crab *Callinectes sapidus* reveals a novel member of the PDH neuropeptide family. *Biochem. Biophys. Res. Commun.* 205 410–416. 10.1016/0305-0491(95)00126-3 7999056

[B35] Kleinholz L. H. (1975). Purified hormones from the crustacean eyestalk and their physiological specificity. *Nature* 258 256–257. 10.1038/258256a0 1202356

[B36] Lear B. C. Merrill C. E. Lin J. M. Schroeder A. Zhang L. Allada R. (2005). A G protein-coupled receptor, groom-of-PDF, is required for PDF neuron action in circadian behaviour. *Neuron* 48 221–227. 10.1016/j.neuron.2005.09.008 16242403

[B37] Li L. Floyd P. D. Rubakhin S. S. Romanova E. V. Jing J. Alexeeva V. Y. (2011). Cerebrin prohormone processing, distribution and action in *Aplysia californica*. *J. Neurochem.* 77 1569–1580. 10.1046/j.1471-4159.2001.00360.x 11413240

[B38] Livak K. J. Schmittgen T. D. (2001). Analysis of relative gene expression data using real-time quantitative PCR and the 2^–Δ^ ^Δ^ ^C^_*T*_ method. *Methods* 25 402–408. 10.1006/meth.2001.1262 11846609

[B39] Löhr J. Klein J. Webster S. G. Dircksen H. (1993). Quantification, immunoaffinity purification and sequence analysis of a pigment-dispersing hormone of the shore crab, *Carcinus maenas*. *Comp. Biochem. Physiol. B* 104 699–706. 10.1016/0305-0491(93)90200-o 8472537

[B40] Ma M. Bors E. K. Dickinson E. S. Kwiatkowski M. A. Sousa G. L. Henry R. P. (2009). Characterization of the *Carcinus maenas* neuropeptidome by mass spectrometry and functional genomics. *Gen. Comp. Endocrinol.* 161 320–334. 10.1016/j.ygcen.2009.01.015 19523386 PMC2888039

[B41] Mangerich S. Keller R. (1988). Localization of pigment-dispersing hormone (PDH) immunoreactivity in the central nervous system of *Carcinus maenas* and *Orconectes limosus* (Crustacea), with reference to FMRFamide immunoreactivity in *O. limosus*. *Cell Tiss. Res.* 253 199–208. 10.1007/BF00221755 3416337

[B42] Mangerich S. Keller R. Dircksen H. Ranga Rao K. Riehm J. P. (1987). Immunocytochemical localization of pigment-dispersing hormone (PDH) and its coexistence with FMRFamide-immunoreactive material in the eyestalks of the decapod crustaceans *Carcinus maenas* and *Orconectes limosus*. *Cell Tiss. Res.* 250 365–375. 10.1007/BF00219081

[B43] Marco H. Verlinden H. Vanden Broeck J. Gäde G. (2017). Characterization and pharmacological analysis of a crustacean G protein-coupled receptor: the red-pigment concentrating hormone receptor of *Daphnia pulex*. *Sci. Rep.* 7:6851. 10.1186/1472-6793-10-14 28761110 PMC5537346

[B44] Mayer G. Hering L. Stosch J. M. Stevenson P. A. Dircksen H. (2015). Evolution of pigment-dispersing factor neuropeptides in Panarthropoda: insights from Onychophora (velvet worms) and Tardigrada (water bears). *J. Comp. Neurol.* 523 1865–1885. 10.1002/cne.23767 25722044

[B45] Meelkop E. Marco H. G. Janssen T. Temmerman L. Vanhove M. P. M. Schoofs L. (2012). A structural and functional comparison of nematode and crustacean PDH-like sequences. *Peptides* 34 74–81. 10.1016/j.peptides.2011.11.008 22115566

[B46] Meelkop E. Temmerman L. Schoofs L. Janssen T. (2011). Signalling through pigment dispersing hormone-like peptides in invertebrates. *Prog. Neurobiol.* 93 125–147. 10.1016/j.pneurobio.2010.10.004 21040756

[B47] Mertens I. Vandingenen A. Johnson E. C. Shafer O. T. Li W. Trigg J. S. (2005). PDF receptor signaling in *Drosophila* contributes to both circadian and geotactic behaviors. *Neuron* 48 213–219. 10.1016/j.neuron.2005.09.009 16242402

[B48] Mirabeau O. Joly J. S. (2013). Molecular evolution of peptidergic signalling systems in bilaterians. *Proc. Natl. Acad. Sci. U.S.A.* 110 2028–2037. 10.1073/pnas.1219956110 23671109 PMC3670399

[B49] Ohira T. Nagasawa H. Aida K. (2002). Molecular cloning of cDNAs encoding two pigment-dispersing hormones and two corresponding genes from the kuruma prawn (*Penaeus japonicus*). *Mar. Biotechnol.* 4 463–470. 10.1007/s10126-002-0042-9 14961239

[B50] Oliphant A. Alexander J. L. Swain M. T. Webster S. G. Wilcockson D. C. (2018). Transcriptomic analysis of crustacean neuropeptide signaling during the moult cycle in the green shore crab, *Carcinus maenas*. *BMC Genomics* 19:711. 10.1186/s12864-018-5057-3 30257651 PMC6158917

[B51] Ou J. Deng H.-M. Zheng S.-C. Huang L.-H. Feng Q.-L. Liu L. (2014). Transcriptomic analysis of developmental features of *Bombyx mori* wing disc during metamorphosis. *BMC Genomics* 15:820. 10.1186/1471-2164-15-820 25261999 PMC4196006

[B52] Powell B. L. (1962). Types, distribution and rhythmical behaviour of the chromatophores of juvenile *Carcinus maenas* (L.). *J. Anim. Ecol.* 31 251–261. 10.1007/BF00390898

[B53] Rao K. R. (2001). Crustacean pigment-effector hormones: chemistry and functions of RPCH, PDH and related peptides. *Am. Zool.* 41 364–379. 10.1093/icb/41.3.364 31919651

[B54] Rao K. R. Mohrherr C. J. Riehm J. P. Zahnow C. A. Norton S. Johnson L. (1987). Primary structure of an analog of crustacean pigment-dispersing hormone from the lubber grasshopper *Romalea microptera*. *J. Biol. Chem.* 262 2672–2675. 3818616

[B55] Rao K. R. Riehm J. P. (2001). Pigment-dispersing hormones. *Ann. N. Y. Acad. Sci.* 680 78–88. 10.1111/j.1749-6632.1993.tb19676.x 8512238

[B56] Renn S. C. Park J. H. Rosbash M. Hall J. C. Taghert P. H. (1999). A pdf neuropeptide gene mutation and ablation of PDF neurons each cause severe abnormalities of behavioral circadian rhythms in *Drosophila*. *Cell* 99 791–802. 10.1016/s0092-8674(00)81676-1 10619432

[B57] Saver M. A. Wilkens J. L. Syed N. I. (1999). In situ and *in vitro* identification of cardiac ganglion neurons in the crab *Carcinus maenas*. *J. Neurophysiol.* 81 2964–2976. 10.1152/jn.1999.81.6.2964 10368413

[B58] Schürmann F.-W. Sandeman R. Sandeman D. (1991). Dense-core vesicles and non-synaptic exocytosis in the central body of the crayfish brain. *Cell Tiss. Res.* 265 493–501. 10.1007/BF00340872

[B59] Semmens D. C. Mirabeau O. Moghul I. Pancholi M. R. Wurm Y. Elphick M. R. (2016). Transcriptomic identification of starfish neuropeptide precursors yields new insights into neuropeptide evolution. *Open Biol.* 6:150224. 10.1098/rsob.150224 26865025 PMC4772807

[B60] Sonnhammer E. L. L. von Heijne G. Krogh A. (1988). “A hidden Markov model for predicting transmembrane helices in protein sequences,” in *Proceedings of the Sixth International Conference on Intelligent Systems for Molecular Biology*, eds Glasgow J. Littlejohn T. Major F. Lathrop R. Sankoff D. Sensen C. (Menlo Park, CA: AAAI Press), 175–182. 9783223

[B61] Stefanini M. De Martino C. Zamboni L. (1967). Fixation of ejaculated spermatozoa for electron microscopy. *Nature* 216 173–174. 10.1038/216173a0 4862079

[B62] Strauss J. Dircksen H. (2010). Circadian clocks in crustaceans: identified neuronal and cellular systems. *Front. Biosci.* 15:1040–1074. 10.2741/3661 20515741

[B63] Strauss J. Zhang Q. Verleyen P. Huybrechts J. Neupert S. Predel R. (2011). Pigment-dispersing hormone in *Daphnia* interneurons, one type homologous to insect clock neurons displaying circadian rhythmicity. *Cell. Mol. Life Sci.* 68 3403–3423. 10.1007/s00018-011-0636-3 21365282 PMC11115014

[B64] Sullivan J. M. Genco M. C. Marlow E. D. Benton J. L. Beltz B. S. Sandeman D. C. (2009). Brain photoreceptor pathways contributing to circadian rhythmicity in crayfish. *Chronobiol. Int.* 26 1136–1168. 10.3109/07420520903217960 19731110 PMC3072780

[B65] Tran N. M. Mykles D. L. Elizur A. Ventura T. (2019). Characterization of G-protein coupled receptors from the blackback land crab *Gecarcinus lateralis* Y organ transcriptome over the molt cycle. *BMC Genomics* 20:74. 10.1186/s12864-018-5363-9 30669976 PMC6341585

[B66] Veenstra J. A. (2010). Neurohormones and neuropeptides encoded by the genome of *Lottia gigantea*, with reference to other mollusks and insects. *Gen. Comp. Endocrinol.* 167 86–103. 10.1016/j.ygcen.2010.02.010 20171220

[B67] Veenstra J. A. (2011). Neuropeptide evolution: neurohormones and neuropeptides predicted from the genomes of *Capitella teleata* and *Helobdella robusta*. *Gen. Comp. Endocrinol.* 171 160–175. 10.1016/j.ygcen.2011.01.005 21241702

[B68] Veenstra J. A. (2016). Similarities between decapod and insect neuropeptidomes. *PeerJ.* 4:e2043. 10.7717/peerj.2043 27257538 PMC4888303

[B69] Verde M. A. Barriga-Montoya C. Fuentes-Pardo B. (2007). Pigment dispersing hormone generates a circadian response to light in the crayfish, *Procambarus clarkii*. *Comp. Biochem. Physiol. A* 147 983–992. 10.1016/j.cbpa.2007.03.004 17428715

[B70] Webster S. G. (1998). Peptidergic neurons in barnacles: an immunohistochemical study using antisera raised against crustacean neuropeptides. *Biol. Bull.* 195 282–289. 10.2307/1543140 28297614

[B71] Webster S. G. Wilcockson D. C. Mrinalini Sharp J. H. (2013). Bursicon and neuropeptide cascades during the ecdysis program of the shore crab, *Carcinus maenas*. *Gen. Comp. Endocrinol.* 182 54–64. 10.1016/j.ygcen.2012.11.018 23247273

[B72] Wilcockson D. C. Zhang L. Hastings M. H. Kyriacou C. P. Webster S. G. (2011). A novel form of pigment-dispersing hormone in the central nervous system of the intertidal marine isopod, *Eurydice pulchra* (Leach). *J. Comp. Neurol.* 519 562–575. 10.1002/cne.22533 21192084

