# Supplementary figures and images for: Pigment Dispersing Factors and Their Cognate Receptors in a Crustacean Model, With New Insights Into Distinct Neurons and Their Functions
Source: Front Neurosci. 2020 Oct 29;14:595648. doi: 10.3389/fnins.2020.595648 (PMC7658428; doi:10.3389/fnins.2020.595648)

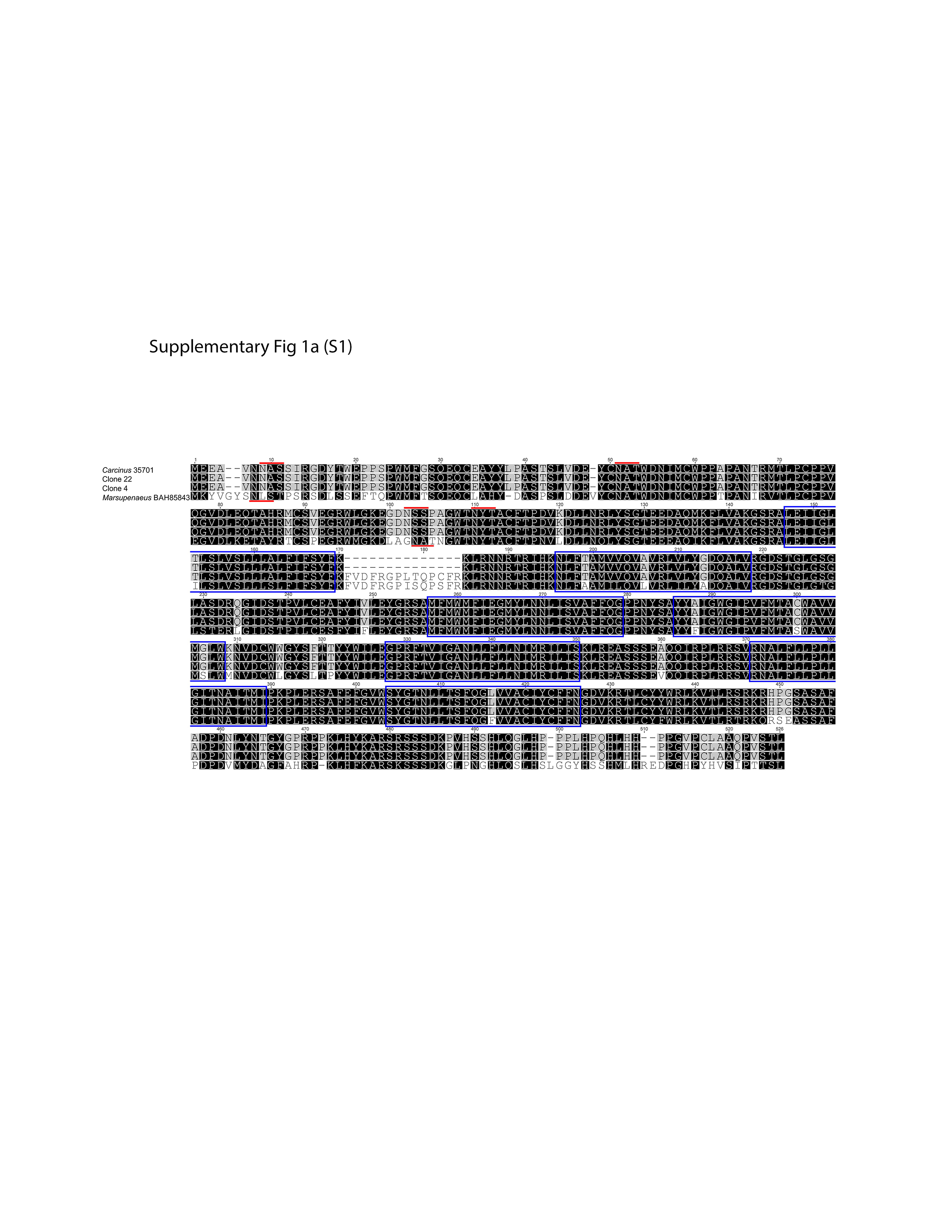

Supplement: Supplementary Figure 1 — (A) Amino acid alignments C. maenas PDHR-like 35701 and Marsupenaeus japonicus AB778163. Amino acid alignment of C. maenas 35701, from transcriptome, and clones identified by PCR and Sanger sequencing, compared to a putative PDHR from Marsupenaeus japonicus (AB 478163) highlighting identical (black)/similar (gray) boxed amino acid residues. Gaps added to maximize sequence identity. The seven predicted transmembrane domains are highlighted in blue, six conserved extracellular N-terminal domain cysteines are marked by asterisks, and putative N-glycosylation sites on the extracellular N-terminal domain are indicated by red lines. (B) Amino acid alignments C. maenas PDHR-like 35701, PDHR 41189, and 43673 [file Image_1.JPEG]

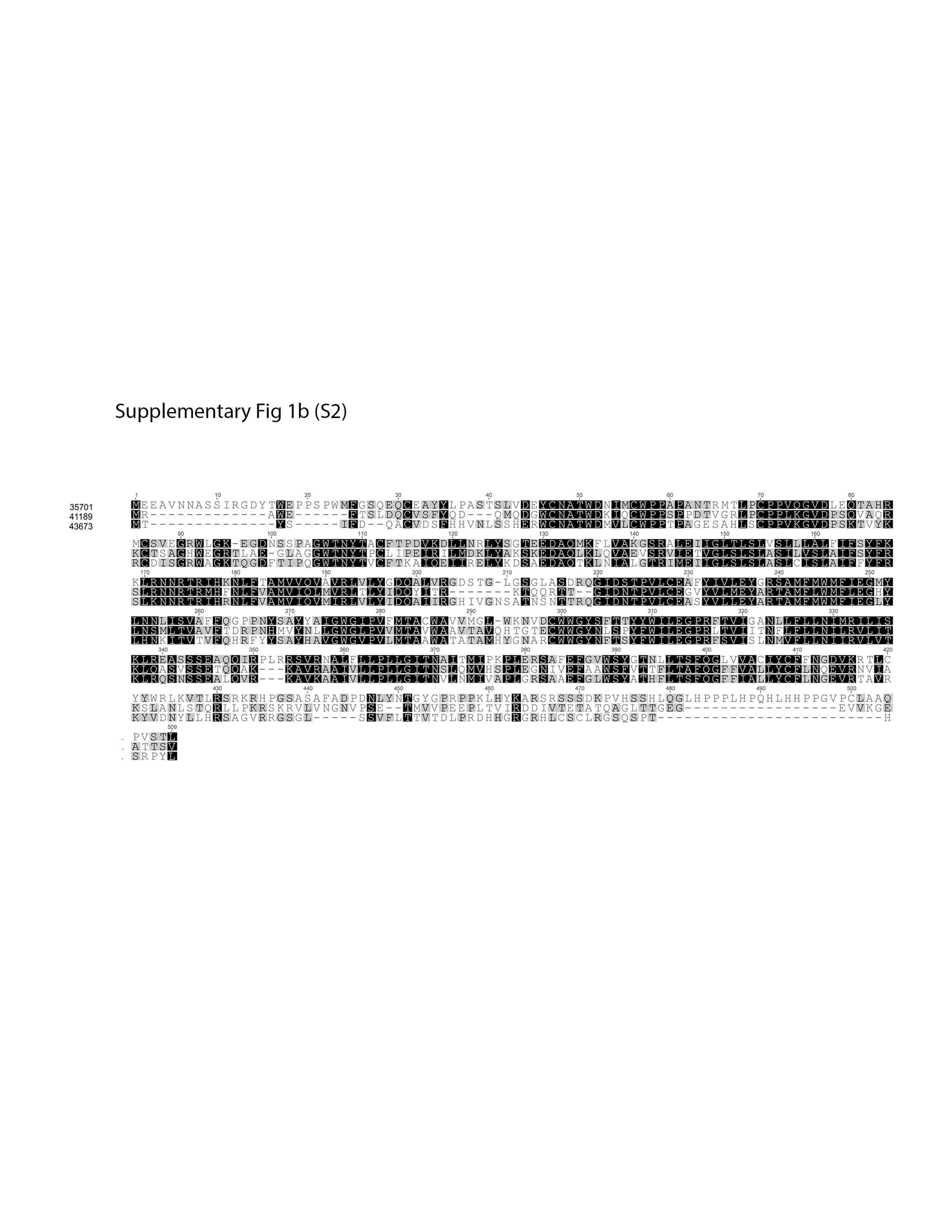

Supplement: Supplementary file 2 [file Image_2.JPEG]
