# Supplementary material for: Pigment Dispersing Factors and Their Cognate Receptors in a Crustacean Model, With New Insights Into Distinct Neurons and Their Functions
Source: Front Neurosci. 2020 Oct 29;14:595648. doi: 10.3389/fnins.2020.595648 (PMC7658428; doi:10.3389/fnins.2020.595648)
Supplement: Supplementary Table 1 — Primers used. [file Table_1.DOCX]

Table 1. Supplementary

Abbreviations: T7: adapter sequence TAATACGACTCACTATAGGGAGA

| Target and method | Forward primer | Reverse primer | Taqman probe | Amplicon  Size (bp) |
| --- | --- | --- | --- | --- |
|  |  |  |  |  |
| PDHR 43673 Expression | CACCTCTCCGCCCATGACTTACTCC | AGCCGCCCCAACTGTCATAAAGTACG |  | 1381 |
| PDHR 41189 Expression | CACCATGAGGGCGTGGGAGTTTAC | TGGAGGGTCATACAGAGGTTGTTGC |  | 1372 |
| PDHR 35701 Expression | CACCATGGAGGAGGCCGTC | GTCATAAGGTGGAAACTGGCTGG |  | 1525 |
| PDHR 43673 Standard | GGCCTGCGTCGATTCCTT | ATGGCCTTGGTGAAACAGAC |  | 264 |
| PDHR 43673 Taqman | GGCCTGCGTCGATTCCTT | TGCACCACCGCTCATGAG | FAM-ACCACGTCAATCTT | - |
| PDHR 41189 Standard | GCCCTCGTCTCACCGTAATA | CTTGGAAGGCAGTGAGGAAG |  | 252 |
| PDHR 41189 Taqman | TGAGAGCTGCCATCGTGTTG | CCATCTGCAGGCTGTTTGTG | FAM-TGCCACTCCTGGGCA | - |
| Ubiquitin-conjugating  Enzyme E2 L3 (UbcE2)  Standard | ACATTCGAAGGTCTGGCATC | GAACTTCTTGCGGTCTTTGG |  | 371 |
| Ubiquitin-conjugating  Enzyme E2 L3 (UbcE2)  Taqman | TCACCTGGCAGGGACTCATT | CCTGAACGCTCCCTTGTTGT | FAM-ACCCGAGAACCCACC | - |
| Elongation factor 1a  (Elf1a) Standard | CCAAGATCGAGCGTAAGAGC | CGATCACCTGAGCTGTGAAA |  | 396 |
| Elongation factor 1a  (Elf1a) Taqman | GAGCGGCAGCTATGAGTTCAT | TGGATGGAGGCTCAATGTTG | VIC-CTCTCTTTGACGCTCTGG | - |
| PDH-1 endpoint PCR | CGCAGCAGATATACCGTGTG | TACATGAGAGGCCGACGTTT |  | 320 |
| PDH-2 endpoint PCR | ACCTGTCTACCTACCTACCTG | CCGAGCAGTGAGTTGATGAG |  | 261 |
| PDH-3 endpoint PCR | TTACAGCGTGGTCGTAGCTG | AAGGTGGAAGAGACCATAATGAA |  | 309 |
| e-PDH endpoint PCR | ATCCTGTCGTGTGCATACCA | TAAGCCCATCGTTGAGGAGC |  | 329 |
| PDH-1 In situ | CGCAGCAGATATACCGTGTG | T7-TACATGAGAAGGCCGACGTTT |  | 343 |
| PDH-2 In situ | ACCTGTCTACCTACCTACCTG | T7-GCCTGCTTGGAGTCATCATCC |  | 312 |
| PDH-3 In situ | TTACAGCGTGGTCGTAGCTG | T7-AAGGTGGAAGAGACCATAATGAA |  | 332 |
| e-PDH In situ | ATCCTGTCGTGTGCAATACCA | T7-TAAGCCCATCGTTGAGGAGC |  | 353 |
| PDH-2 5’ RACE | CGACTGGAGCACGAGGACACTGA | TGGAGAGGCCGAGCAGTGAGT |  | 347 |
| PDH-2 5’ RACE nest | GGACACTGACATGGACTGAAGGAGTA | As above |  | 333 |
| PDH-3 5’ RACE | As above | ACTTCAGGATGTGTGCCGCCAGGT |  | 246 |
| PDH-3 5’ RACE nest | “ | ATGTGTGCCGCCAGGTTAGCCAAG |  | 224 |
| e-PDH 5’ RACE | “ | GTCCCTGAAATTAACGTCTGCCACG |  | 424 |
| e-PDH 5’ RACE nest | “ | CCAGCATTCCTCATGTTTCCGAG |  | 390 |
| PDH-2 3’ RACE | TGAAGGTGATCCACGCCCCGCAGGAGGCT | GGCTGTCAACGATACGCTACGTAACG |  | 245* |
| PDH-2 3’ RACE nest | GCTGGAGGCTGCCGCAGGTCTCGCACACA | CGCTACGTAACGGCATGACAGTG |  | 226* |
| PDH-3 3’ RACE | TCCGTCCTCTTCACCCAGGGACAG | As above |  | 301* |
| PDH-3 3’ RACE nest | TAACCTGGCGGCACACATCCTGAA | “ |  | 245* |
| e-PDH 3’ RACE | GACCGTCATCTCCCACCCCACTTG | “ |  | 895* |
| e-PDH 3’ RACE nest | ACCTTCGCTCTGCCCACCACACTC | “ |  | 797* |

- Amplicon lengths indicated for 3’ RACE PCRs exclude the polyA tail.
